# IL-6 and TNFα Drive Extensive Proliferation of Human Tregs Without Compromising Their Lineage Stability or Function

**DOI:** 10.3389/fimmu.2021.783282

**Published:** 2021-12-23

**Authors:** Nikolaos Skartsis, Yani Peng, Leonardo M. R. Ferreira, Vinh Nguyen, Emilie Ronin, Yannick D. Muller, Flavio Vincenti, Qizhi Tang

**Affiliations:** ^1^ Department of Surgery, University of California San Francisco, San Francisco, CA, United States; ^2^ Division of Nephrology, Department of Medicine, University of California San Francisco, San Francisco, CA, United States; ^3^ Diabetes Center, University of California San Francisco, San Francisco, CA, United States

**Keywords:** Tregs, inflammation, IL-6, TNFα, CD28 signaling, metabolomics, GVHD

## Abstract

Treg therapies are being tested in clinical trials in transplantation and autoimmune diseases, however, the impact of inflammation on Tregs remains controversial. We challenged human Tregs *ex-vivo* with pro-inflammatory cytokines IL-6 and TNF*α* and observed greatly enhanced proliferation stimulated by anti-CD3 and anti-CD28 (aCD3/28) beads or CD28 superagonist (CD28SA). The cytokine-exposed Tregs maintained high expression of FOXP3 and HELIOS, demethylated FOXP3 enhancer, and low IFNγ, IL-4, and IL-17 secretion. Blocking TNF receptor using etanercept or deletion of *TNF receptor 2* using CRISPR/Cas9 blunted Treg proliferation and attenuated FOXP3 and HELIOS expression. These results prompted us to consider using CD28SA together with IL-6 and TNF*α* without aCD3/28 beads (beadless) as an alternative protocol for therapeutic Treg manufacturing. Metabolomics profiling revealed more active glycolysis and oxidative phosphorylation, increased energy production, and higher antioxidant potential during beadless Treg expansion. Finally, beadless expanded Tregs maintained suppressive functions *in vitro* and *in vivo*. These results demonstrate that human Tregs positively respond to proinflammatory cytokines with enhanced proliferation without compromising their lineage identity or function. This property can be harnessed for therapeutic Treg manufacturing.

## Introduction

Tregs are a subset of CD4^+^ T cells that prevent unwanted immune activation in steady state and promote resolution of immune response at the site of inflammation (1–3). In mouse models of type 1 diabetes, Tregs isolated from inflamed islets, but not from lymphoid organs, are able to suppress anti-islet autoimmune attacks (4). In humans, higher FOXP3 in graft biopsies predicts reversibility of T cell-mediated rejection of kidney transplant whereas higher Tregs in the tumor microenvironment is associated with worse prognosis (5, 6). However, it has been suggested that Tregs may be destabilized under certain inflammatory conditions and there has been debate on the impact of proinflammatory signals on Treg function (7). During immune activation, proinflammatory cytokines such as TNF*a* and IL-6 are produced by macrophages, neutrophils, T cells, B cells, and stromal cells (8–11). *a* and IL-6 promote T cell activation (12, 13) and increase resistance to Treg-mediated suppression (14–16), both of these functions contribute to mounting an effective immune response. While there is consensus that TNF*a* and IL-6 heighten effector T cell (Teff) resistance to suppression by Tregs (17, 18), their direct impact on Tregs, especially on human Tregs, is unclear.

The effect of TNF*a* on mouse Tregs has been extensively studied. While TNF*a* impairs the differentiation and function of TGFβ-induced Tregs (19), it is generally accepted that TNF*α* exerts positive effects on thymic derived Tregs (20–22). TNF*α* optimally activates Tregs *via* upregulating TNFR2, 4-1BB, and OX40 expression (23, 24). TNFR2-deficient Tregs lose Foxp3 expression and cannot protect against colitis when co-transferred with naïve CD4^+^ T cells into Rag1^KO^ mice (25). TNFR2-expressing Tregs are critical to suppressing EAE by limiting T cell activation in the CNS during overt inflammation (26). Finally, TNF*a*-primed mouse Tregs have enhanced efficacy against graft-versus-host disease (GVHD) when compared with unprimed Tregs (27, 28). Overall, these results suggest that TNFα negatively affects TGFβ-induced Tregs, but exerts a positive effect on thymic derived Tregs in mice.

The effects of TNF*a* on human Tregs has been more controversial. Tregs isolated from rheumatoid arthritis patients have been reported to lose FOXP3 expression and convert into pathogenic T cells when exposed to TNF*a in vitro* (29, 30). TNFα partially abrogated the suppressive function of CD4^+^CD25^+^ T cells isolated from chronic HBV-infected patients (31). Activation of the canonical NF-κB pathway in CD45RA^-^ Tregs by TNF*a*-TNFR2 interaction downmodulated their suppressive function (32). However, these findings have been challenged by others who report a pro-regulatory role of TNF*a* in optimally activating Tregs isolated from healthy donors, without negatively affecting their *in vitro* suppressive function (33). Similar to studies in mouse Tregs, TNFR2 expression identifies a subset of highly suppressive Tregs isolated from PBMC (34). TNFR2 signaling has been shown to enhance human Treg proliferation in response to IL-2 *via* activation of the non-canonical NF-κB pathway (35). As a result, TNFR2 agonists are used in *ex-vivo* human Treg expansion protocols to facilitate homogeneous high purity cellular products (36, 37). Multiple studies have shown that TNFα blockade therapy paradoxically exacerbate autoimmune pathologies (38, 39), suggesting a regulatory role of TNF*a* in these disease settings.

Multiple studies reported a negative role of IL-6 on mouse Tregs. IL-6 prevents the differentiation of naïve CD4^+^ T cells into peripherally induced Tregs in a dominant and non-redundant manner (40). IL-6 has been reported to destabilize mouse thymic derived Tregs and drive them to produce IL-17 *in vitro* (41). However, Tregs isolated from inflamed CNS lesions in EAE-affected mice are insensitive to IL-6-driven Th17 conversion due to a downregulation of IL-6R*a* and gp130 (42). IL-6 has been found to increase the rate of Foxp3 proteasomal degradation *via* downregulation of the expression of deubiquitinase USP7, which is normally upregulated and associated with Foxp3 in the nucleus of Tregs (43). In contrast, IL-6 Tg mice harbor increased numbers of Tregs in their lymphoid organs with intact functional capacity against naïve T cells *in vitro* (44). While IL-6 enhances the generation of Th17 cells, classic signaling *via* IL-6Ra induces double positive RORγt^+^Foxp3^+^ Tregs with potent immunoregulatory properties in a mouse model of glomerulonephritis (45). The prevailing view is that IL-6 negatively impacts mouse Tregs, but emerging data suggest that differential mode of signaling (classic versus trans-signaling) may have divergent impacts on mouse Tregs.

Similar to findings in mouse Tregs, IL-6 prevents *in-vitro* induction of human Tregs from conventional T cells (Tconvs) (46). Peripheral blood CD4^+^CD25^+^ Tregs downregulate FOXP3 and lose their suppressive function following IL-6 exposure (47). Retinoic acid downregulates IL-6R*a* and renders Tregs insensitive to the destabilizing effect of IL-6 (47). gp130^hi^ Tregs are enriched amongst naïve CD45RA^+^ Tregs isolated from human PBMC and have lower *in vitro* suppressive capacity when compared to gp130^lo^ Tregs (48). Another group has reported the presence of a subpopulation of human IL-6R*a* ^hi^CD45RA^-^ Tregs in PBMC that have potent suppressive capacity *in vitro*, demethylated Treg-specific demethylated region (TSDR) and a Th_17_ transcriptional signature (49). These studies highlight the heterogeneity of circulating human Tregs. However, there is paucity of studies measuring the direct impact of IL-6 on human Tregs.

In this study, we examined the direct impact of TNF*a* and IL-6 on human Tregs in an ex-vivo culture system and evaluated the *in-vivo* function of TNF*a* and IL-6 exposed human Tregs in a xenogeneic GVHD model in humanized mice. Our data show a positive role of both IL-6 and TNF*a* on Tregs in promoting their proliferation without lineage destabilization, suggesting that Tregs can respond to proinflammatory signals to increase their presence at sites of inflammation without compromising their lineage stability.

## Materials and Methods

### Human Peripheral Blood Samples

De-identified peripheral blood units were purchased from StemCell Technologies (Vancouver, Canada). Peripheral blood samples from 25 healthy donors (11 male and 14 female) between the age of 21 and 72 were used.

### *Ex-Vivo* Treg Culture

Human blood samples were processed the same day after collection using ficoll (Cytiva, Marlborough MA) density gradient to isolate PBMC. PBMC were stained with anti-CD4 FITC (clone OKT4), anti-CD25 APC (clone 4E3) and anti-CD127 PE (clone HIL-7R-M21, all from BD Biosciences, San Jose, CA). CD4^+^CD25^+^CD127^lo/-^ Tregs and CD4^+^CD25^-^CD127^+^ Tconvs were purified using FACS. Post sort analyses confirmed >99% purity. Purified Tregs were plated in 48-well plate at a density of 1x10^5^ cells/well in 250μL medium consisting of 10% heat-inactivated fetal bovine serum (Biosource International), nonessential amino acids, 0.5 mM sodium pyruvate, 5 mM Hepes and 1 mM glutaMax I (all from Invitrogen) in an RPMI-1640 base. Human anti-CD3 and anti-CD28-conjugated dynabeads (aCD3/CD28 beads; Fischer Scientific, Hampton, NH) were added to the T cells at 1:1 bead to cell ratio. Alternatively, CD28 superagonist (CD28SA) (5mg/mL; clone anti-CD28.1; Ancell, Stillwater, MN) were used to stimulate the T cells. Recombinant human IL-2 (either 300IU/mL or 15IU/mL, as indicated in the result section; Proleukin, Prometheus laboratories, Switzerland) were added at concentration indicated. Recombinant human IL-6 (15 to 150ng/ml as indicated in the result section; Peprotech, Rocky Hill, NJ) and recombinant human TNF*a* (50ng/ml; Peprotech, Rocky Hill, NJ) were also added in selected wells. Etanercept (5μg/mL; ETN-Enbrel, Pfizer) was added in selected experiments. Ex-vivo expansion cultures were supplemented with fresh media containing IL-2 on day 0, 2, 5, and every other day thereafter, until the cells returned quiescent state (typically, day 9 for aCD3/CD28 bead-stimulated Tregs and day 14 for CD28SA stimulated Tregs). IL-6 and TNFα were supplemented on days 0, 2, and 5 of culture. aCD3/28 bead stimulated CD4^+^ Tconvs were included in some experiments as a reference cell population.

### Flow Cytometry

Single-cell suspensions were recovered from ex-vivo expanded Treg cultures. The cellular suspensions were first stained with surface antibodies to CD4 (Per-CP; clone SK3), CD25 (PE-Cy7; clone 4E3), TNFR2 (APC; clone hTNFR-M1), and fixable viability dye (APC-e780, all from purchased from BD Biosciences) prior to fixation and permeabilization using kit according to manufacturer protocol (catalog number 88-8824-00; Thermofisher) and intracellular staining for FOXP3 (e450; clone PCH101) and HELIOS (FITC; clone 22F6, both from BD Biosciences). The samples were analyzed on a FACS LSR-II flow cytometer (BD Biosciences) and analyzed using FACSDiva (BD Biosciences) or Flow Jo software (Tree Star, Ashland, OR).

### *In-Vitro* Treg Suppression Assay

Ex-vivo expanded human Tregs were harvested on day 7 of culture and varying numbers of Tregs were then co-cultured with 1x 10^5^ ex-vivo expanded Tconv at Treg/Tconv ratios of 1:1, 1:2, 1:4 and 1:8 in the presence of aCD3/CD28 beads (1:10 bead to Tconv ratio). 0.5μCi [^3^H]- Thymidine was added 16hrs before the cells were harvested for analysis on day 4 of co-culture. Triplicate wells were established for each condition.

### TSDR Analysis

*Ex-vivo* expanded Tregs (2x10^6^ from each condition) were harvested on day 7 of culture and then cryopreserved in FBS containing 10% DMSO at -80°C. The samples were submitted to Epigendx (Boston, MA) for analysis of DNA methylation of the TSDR in FOXP3 intron 1 using the ADS783FS2 assay.

### Cytokine Assay

Supernatants of Treg and Tconv cultures were aspirated on day 7 and sent for analysis using a 42-plexed Luminex human cytokine detection kit (EveTechnologies, Vancouver, Canada). The cytokine concentrations were normalized per 10^6^ cells for each condition. For all culture conditions, Cells were plated at 1x10^5^/well in one well of 48-well plates in 250mL medium supplemented with cytokines as indicated; culture media were doubled on day 2; then cells were counted and resuspended at 2.5x10^5^ cells per 1mL on day 5; and the supernatant was collected on day 7 for multiplex Luminex analysis on day 7 and the cell counts on day 7 were used to normalize the cytokine concentrations.

## Humanized NSG Mouse Graft-Versus-Host-Disease Models

Eight to twelve-week-old NOD.Cg-Prkdc^scid^Il2rg^tm1Wjl^/SzJ (NSG, Jackson Laboratory, Stock No 005557) male mice were bred in our animal facilities in specific pathogen-free conditions. To induce GVHD, NSG mice were irradiated (2.5Gy) one day prior to retroorbital vein infusion of either 4 x10^6^ fresh PBMCs or ex-vivo expanded Tconvs. In GVHD prevention experiments, some mice receive co-infusion of ex-vivo expanded Tregs. In GVHD treatment experiments, Tregs were infused 4 days after PBMC infusion. Xenogeneic GVHD development was evaluated by clinical examination and body weight measurements for either 60 days (prevention experiments) or 100 days (treatment experiments). To assess the phenotypical stability of *ex vivo* expanded Tregs after adoptive transfer, irradiated NSG mice were injected with 2x10^6^ fresh autologous PBMCs 9 days before receiving 4x10^6^ CFSE-labeled Tregs. On day 5 after Treg infusion, peripheral blood and lung tissue were obtained for analyses using flow cytometry.

### *TNFR2* Gene Deletion Using CRISPR

Ribonucleoprotein complexes were made by complexing crRNAs and tracrRNAs chemically synthesized (Integrated DNA Technologies (IDT), Coralville, IA) with recombinant Cas9NLS (QB3 Macrolab, UC Berkley, CA) as previously described (50). Guide RNA sequences used for gene editing were (1) GGTTCTTGACTACCGTAATT (scrambled gRNA) and (2) GGCAUUUACACCCUACGCCC (*TNFR2* gene). Briefly, Lyophilized RNAs were resuspended at 160μM in 10mM Tris-HCL with 150mM KCL and stored in aliquots at minus 80. The day of electroporation, CrRNA and tracrRNA aliquots were thawed and mixed at a 1:1 volume and incubated 30 minutes at 37°C for annealing. The 80µM guide RNA complex was mixed at 37°C with Cas9 NLs at a 2:1 gRNA to Cas9 molar ratio for another 15 minutes and the mixture is defined as RNP at 20µM. RNP were electroporated using a Lonza 4D 96-well electroporation system (code EH115). Genome editing efficiency was measured by assessing cell surface TNFR2 expression using flow cytometry on days 4 and 8 of culture.

### Metabolomics

At least 2x 10^6^ FACS sorted fresh and ex-vivo expanded Tregs were collected on days 0 and 7 of culture, respectively. The cells were stored according to manufacturer protocol prior to being sent for analysis using capillary electrophoresis- and liquid chromatography- mass spectrometry platforms to generate their global metabolic profile (Human Metabolome Technologies, Boston, MA).

### Statistics

Kaplan-Meier survival graphs were constructed, and a log-rank comparison of the groups was used to calculate p-values. The paired t test was used for comparison of experimental groups. Differences were considered significant for p less than 0.05. Prism software (GraphPad Software, San Diego, CA) version 9 was used for data analysis and graphing data.

### Study Approval

All experiments were approved (IACUC protocol No. AN183959-02) and conducted in accordance with UCSF IACUC regulations.

## Results

### IL-6 and TNFα Promoted Proliferation of Lineage-Committed Tregs

**Figure 1:**
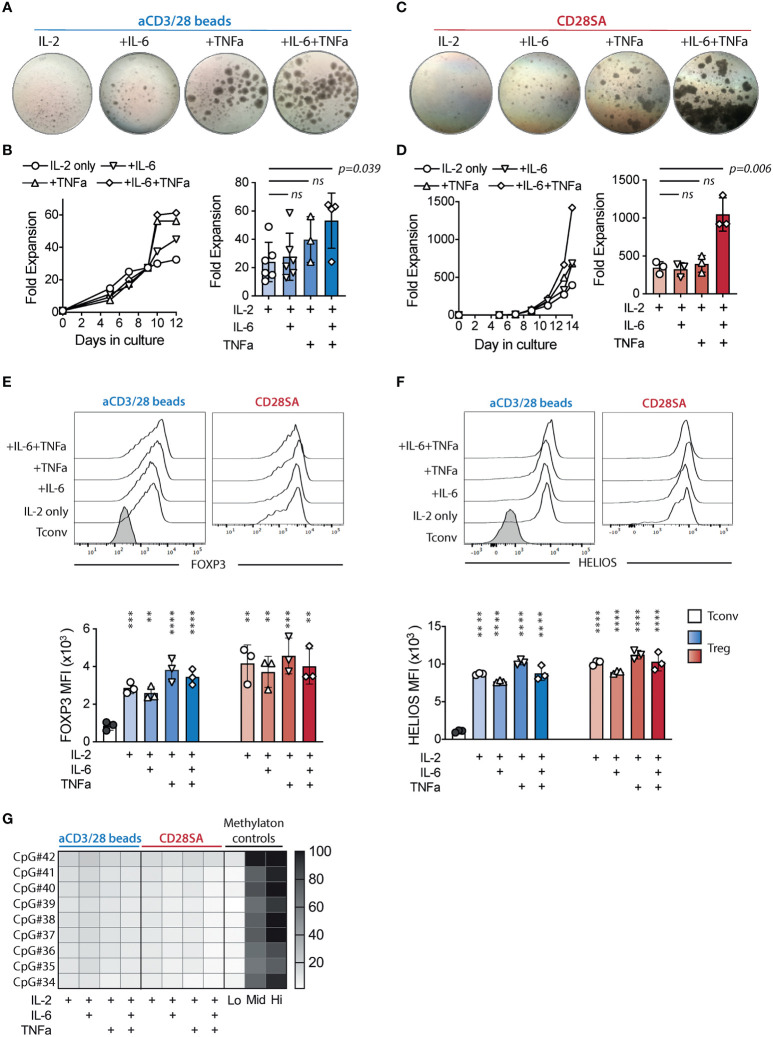
IL-6 and TNF*α* augmented *ex-vivo* proliferation of lineage committed human Tregs. FACS purified human Tregs were stimulated with either aCD3/28 beads or CD28SA and cultured in the presence of 300 IU/ml rhIL-2 with or without TNF*α* and IL-6 for 12 and 14 days as indicated **(A)** Microscopic pictures of aCD3/28 bead-stimulated Treg cultures on day 8 of culture. Original magnification was 10x. **(B)** An example of aCD3/28 bead-stimulated Treg expansion kinetics from one representative experiment (left) and a summary of fold expansion on day 12 of 3 to 6 independent experiments (right) are shown. **(C, D)** Same as panel **(A, B)**, except the Tregs were stimulated with CD28SA and overall expansion (right panel) was assessed on day 14. Statistical significance was assessed using one-way ANOVA and Dunnett’s multiple comparisons posttest using IL-2 alone as reference. *p* values are stated. Results shown used cells from 3 distinct donors and are representative of 6 independent experiments using cells from 6 distinct donors. **(E, F)** Flow cytometric analysis of FOXP3 **(E)** and HELIOS **(F)** expression in Tregs on day 9 after stimulation. Representative histograms (left) and summaries of mean fluorescent intensities (MFI) from 3 independent experiments (right) are shown. Statistical significance was assessed using one-way ANOVA with Geisser-Greenhouse’s correction and Dunnett’s multiple comparisons test using either Tconv or Treg treated with IL-2 alone **(E, F)** as a reference. *p* values are marked as ns, not significant, *p* > 0.05, **p < 0.01, ***p < 0.001, ****p < 0.0001. **(G)** Heatmap summary of TSDR demethylation of Tregs expanded in various conditions. Results shown are averages of Treg cultures using 2 unrelated male donors in 2 independent experiments.

To determine the direct effects of TNFα and IL-6 on human Tregs, we challenged FACS purified CD4^+^CD25^+^CD127^lo/-^ Tregs (sorting strategy shown in **Supplemental Figure 1**) with IL-6 and/or TNFα, during *in vitro* activation. We stimulated the cells with anti-CD3 and anti-CD28 (aCD3/28) beads in the presence of 300 IU/ml recombinant human IL-2 to simulate antigen activation of Tregs. Activated Tregs formed multicellular clusters, which was followed by cell proliferation and return to a single-celled quiescent state by day 9 of culture as previously reported (51). FACS purified CD4^+^CD25^-^CD127^+^ Tconv were also stimulated using the same conditions and proliferated more intensely than Tregs (**Supplemental Figure 2**). The addition of IL-6, TNFα, or both in the Treg cultures resulted in persistent cell clustering (**Figure 1A**). Moreover, combination of IL-6 and TNFα resulted in significantly improved Treg expansion, which mainly manifested during the later stage of culture (**Figure 1B**).

It has been previously reported that CD28 superagonist antibodies (CD28SA) can also induce ex-vivo expansion of human Tregs (52). We thus replaced the aCD3/28 beads with CD28SA and evaluated the impact of IL-6 and TNFα in the context of a different mitogenic stimulation. While CD28SA-stimulated Tregs showed delayed kinetics in proliferation when compared to their aCD3/CD28-stimulated counterparts, the cells proliferated more persistently, resulting in more overall expansion (**Figures 1C, D**). Exposure to both TNF*a* and IL-6 resulted in robust cell clustering over a prolonged period and synergistic enhancement of cell expansion that was far more pronounced than seen with aCD3/CD28 bead-stimulated Tregs (**Figures 1B, D**).

Since FOXP3 had been reported to limit Treg proliferation and metabolism (53), we wondered if the enhanced proliferation in the presence of TNF*a* and IL-6 was due to destabilization of Tregs. We measured the expression of FOXP3 and HELIOS, the two lineage-defining transcription factors for Tregs (54), on days 9 and 14 during the *ex vivo* expansion (**Supplemental Figure 3**). The results showed that Tregs exposed to IL-6 and/or TNF*a* maintained high levels of FOXP3 and HELIOS expression (**Figures 1E, F**). Furthermore, we analyzed TSDR methylation status in Tregs cultured in various conditions. The results showed a high degree of TSDR demethylation in all expanded Tregs (**Figure 1G**), supporting the flow cytometric data to show that the exposure to IL-6 and/or TNF*a* did not alter Treg identity.

**Figure 2:**
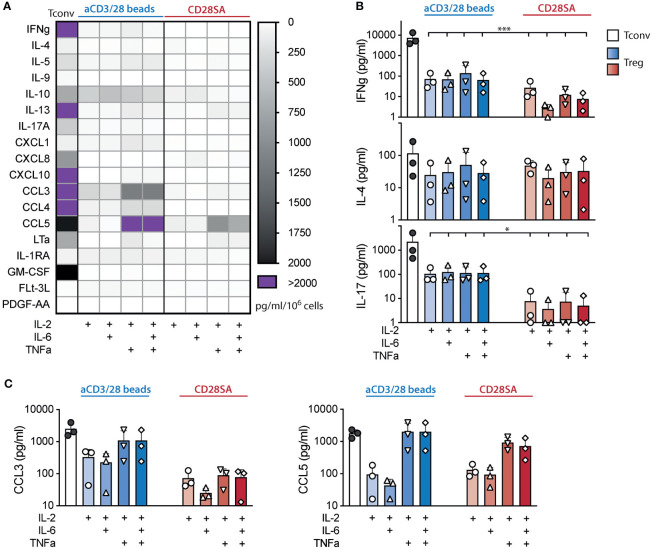
Tregs expanded in IL-6 and TNF*α* did not secrete more proinflammatory cytokines. FACS purified human Tregs were stimulated with either aCD3/28 beads or CD28SA and cultured in the presence of 300 IU/ml rhIL-2 with or without TNF*α* and IL-6 as indicated. Cytokine and chemokine secretion in the culture supernatant of various Treg cultures was assessed using a multiplex Luminex panel. Supernatant of aCD3/28 bead stimulated Tconv cultures is included as a reference. **(A)** Heatmap summary of cytokines and chemokines that were present in any of the culture condition is shown. **(B)** IFNγ, IL-4, and IL-17 concentrations in the Day 7 culture supernatants are shown. **(C)** CCL3 and CCL5 concentrations in the Day 7 culture supernatant are shown. Results shown are summaries of 3 independent experiments using cells from 3 unrelated donors. Statistical significance was assessed using one-way ANOVA with Geisser-Greenhouse’s correction and Dunnett’s multiple comparisons posttest using Treg treated with IL-2 alone **(B, C)** as reference. *p* values are marked as *p < 0.05, ***p < 0.001.

To further assess if IL-6 and/or TNF*a* induced effector functions in Tregs, we analyzed the supernatant of Treg and Tconv cultures on day 7 after stimulation using a 42-plex Luminex assay. Among the 42 cytokines in the panel, 21 were not present in any of the T cell cultures, 3 were added (IL-2, IL-6, and TNF*a*), and 18 were detected in Treg cultures. Among the 18 *de novo* cytokines detected, the amounts found in Treg cultures were markedly lower than those seen in Tconv cultures (**Figure 2A**). Particularly, no increase in IFNγ (Th_1_), IL-4 (Th_2_), or IL-17A (Th_17_) were seen in aCD3/28 bead or CD28SA stimulated cultures by the exposures to IL-6 and/or TNFα (**Figure 2B**). In fact, IL-6 did not induce increased secretion of any of the cytokines. On the other hand, exposure to TNF*a*, alone or in the presence of IL-6, led to a consistent trend of increase of CCL3 and CCL5 (**Figure 2C**). Taken together, these experiments revealed that IL-6 and TNF*a* exposure resulted in robust human Treg proliferation without lineage destabilization.

These results contradicted previous reports on the destabilizing effect of proinflammatory cytokines on Tregs (29, 30, 55, 56). Many of these studies stimulated Tregs in the presence of lower concentration of IL-2 than we used. We hypothesized that the high concentration of IL-2 in our culture condition may have protected Tregs from the destabilizing effect of TNF*a* and IL-6. Therefore, we challenged the Tregs by reducing IL-2 concentration from 300 IU/ml to 15 IU/ml. Treg expansion was minimal with reduced IL-2, even when they were exposed to IL-6 and/or TNFα. FOXP3 expression was reduced when compared to cells cultured in 300 IU/ml of IL-2 but remained significantly elevated when compared to that expressed by similarly expanded Tconvs (**Supplemental Figure 4A**). HELIOS expression was comparably high in Tregs cultured in 15 IU/ml and 300 IU/ml of IL-2 (**Supplemental Figure 4B**). More importantly, Tregs exposed to TNF*a* and IL-6 in the presence of low IL-2 maintained demethylated TSDR (**Supplemental Figure 4C**) and did not produce more effector cytokines (**Supplemental Figure 5A**), including IFNγ, IL-4, or IL-17 (**Supplemental Figure 5B**) when compared to Tregs stimulated without these cytokines. Similar to cultures stimulated in the presence of high IL-2, co-culture with TNFα stimulated higher secretion of CCL5 in Tregs (**Supplementary Figure 5C**). Thus, IL-6 and TNF*a* did not induce Treg destabilization even when IL-2 was limited.

### *Ex-Vivo* Expanded Human Tregs Produce TNFα and Depend on TNFR2 Signaling to Proliferate and Safeguard Their Identity

**Figure 3:**
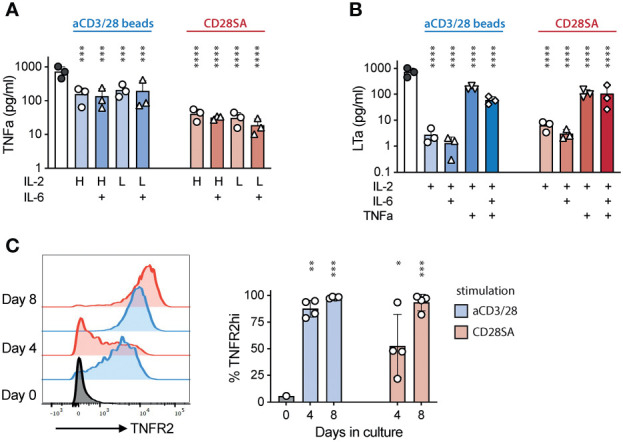
Activated Tregs produced TNF*α* and LT*a* and increased TNFR2 expression. FACS purified human Tregs were stimulated with either aCD3/28 beads or CD28SA and cultured in the presence of 300 IU/mL (H) or 15 IU/mL (L) rhIL-2 with or without IL-6 as indicated. **(A)** TNF*α* and **(B)** LT*a* concentration in the Day 7 culture supernatant of various T cell cultures. **(C)** Flow cytometric analysis of TNFR2 expression in Tregs stimulated with either aCD3/28 beads or CD28SA on day 0, 4 and 8 after stimulation. Representative histograms (left) and summary of percentage of TNFR2^+^ Tregs from 3 independent experiments (right) are shown. Statistical significance was assessed using one-way ANOVA and Dunnett’s multiple comparisons posttest using Treg treated with IL-2 alone **(A, B)** or Day 0 **(C)** as reference. *p* values are marked as ns, not significant, *p < 0.05, **p < 0.01, ***p < 0.001, ****p < 0.0001.

While analyzing cytokine secretion in Treg cultures, we noticed that TNF*α* was consistently detected in all cultures in higher amounts in aCD3/28 bead-stimulated cultures than in CD28SA-stimulated cultures (**Figure 3A**). The amount of TNF*a* in the supernatant was not affected by the concentration of IL-2 or the addition of IL-6. Moreover, another TNF superfamily member, lymphotoxin *a* (LT*a*), was also consistently detected (**Figure 3B**). The presence of TNF*a*, but not IL-6, resulted in significantly increased level of LTa in the aCD3/28 bead-stimulated Treg culture supernatant and a trend of increased LT*a* in CD28SA-stimulated cultures (**Figure 3B**). Both of TNF*a* and LT*a* bind to TNFR1 and TNFR2 (57). It has been well-established that Tregs preferentially express TNFR2 (24). *In vitro* stimulation with either aCD3/28 beads or CD28SA uniformly increased TNFR2 expression, although CD28SA induced upregulation was delayed compared to aCD3/CD28 beads (**Figure 3C**). Thus, activated Tregs produced TNF*a* and LT*a* and also have increased expression of TNFR2, suggesting that TNF*a* and LT*a* may function as autocrine or paracrine factors for activated Tregs.

**Figure 4:**
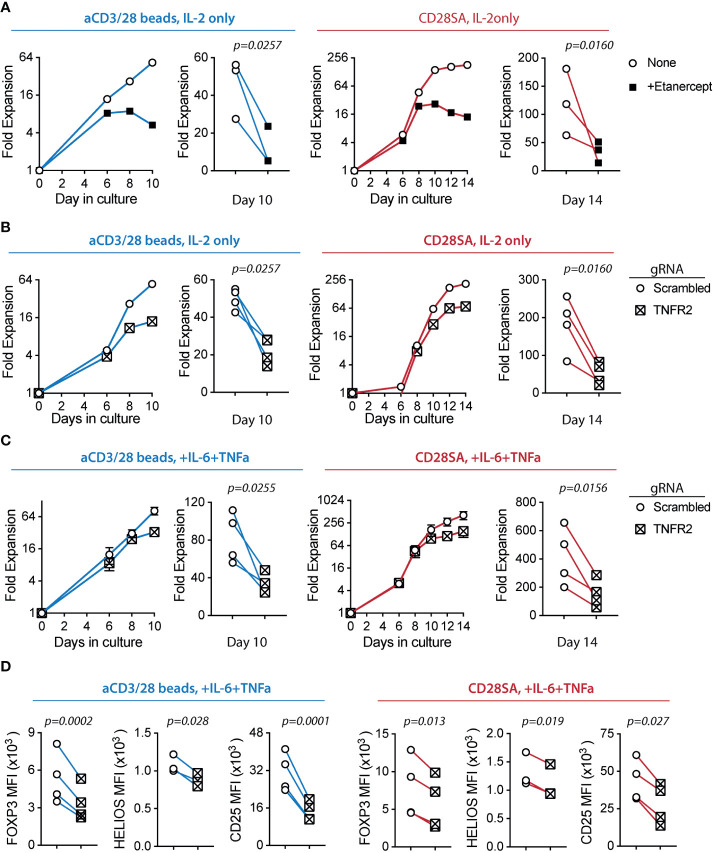
*Ex-vivo* proliferation of human Tregs is dependent on TNFR2 expression. FACS purified human Tregs were stimulated with either aCD3/28 beads or CD28SA and cultured in the presence of 300 IU/ml rhIL-2 with or without TNF*α* and IL-6 for 10 and 14 days, as indicated. **(A)** Representative aCD3/28 bead stimulated Treg expansion kinetics and summary of final fold expansion on day 10 of Treg expanded in the presence or absence of etanercept (left). Similarly, CD28SA stimulated Tregs were expanded for 14 days (right). Results of 3 independent experiments using 3 unrelated donors are shown. **(B)** Tregs were gene edited to delete *TNFR2* gene using CRISPR-Cas9 and then stimulated with either aCD3/28 beads (left) or CD28SA (right). Results shown are from 4 independent experiments using 4 unrelated donors. **(C)** Same as panel **(B)**, except the Tregs were cultured in the presence of IL-6 and TNF*α*. Results shown are from 4 independent donors in 4 independent experiments. **(D)** Tregs were expanded as shown in panel **(C)** and flow cytometric analysis of FOXP3, HELIOS and CD25 expression was performed on day 8 after stimulation. Results show summary of MFI of FOXP3, HELIOS and CD25 from 4 independent donors in 4 independent experiments. Paired t-test was used to determine statistical significance. *p* values are stated.

To investigate a potential role of TNFR2 in human Treg activation, we evaluated the impact of etanercept, a soluble TNFR2-Fc fusion protein, in ex-vivo Treg proliferation. Addition of etanercept significantly reduced Treg proliferation induced by either aCD3/CD28 beads or CD28SA (**Figure 4A**). The inhibitory effect of etanercept was most pronounced during the later stage of Treg culture, suggesting that TNFR2 signaling did not affect initial Treg proliferation likely due to delayed induction of TNFR2 or TNF*a* and LT*a*. Furthermore, etanercept significantly decreased TNFR2 expression and the expanded Tregs showed a trend of moderate reduction in CD25, FOXP3, and HELIOS expression (**Supplemental Figure 6**).

To directly examine the role of TNFR2 in human Treg proliferation, we deleted *TNFR2* gene in purified Tregs using CRISPR/Cas9 technology. On day 4 after activation, only 27.3% of aCD3/CD28 bead stimulated cells electroporated with gRNA targeting the *TNFR2 gene* were TNFR2^+^ when compared to 87.75% of cells that were electroporated with scrambled gRNA (**Supplemental Figure 7A**). Similarly, 14.5% of CD28SA stimulated cells were TNFR2^+^ when compared to 52.6% of cells that were electroporated with scrambled gRNA (**Supplemental Figure 7B**), indicating successful TNFR2 deletion in the majority of Tregs. We observed that TNFR2KO Tregs have significantly reduced ex-vivo expansion with either aCD3/CD28 bead or CD28SA stimulation (**Figure 4B**), demonstrating a positive role of TNFR2 signaling in *ex-vivo* proliferation of human Tregs. It is worth noting that, on day 8 post activation, 44.2% of aCD3/CD28 bead-stimulated and 44.6% of CD28SA-stimulated cells were TNFR2^+^, increased from those detected on day 4 after stimulation. This suggested that the few TNFR2-sufficient Tregs after CRISPR editing had a proliferative advantage over their TNFR2KO counterparts (**Supplemental Figures 7A, B**). Similar to our observation using etanercept, TNFR2KO Tregs showed a trend of decreased expression of CD25, FOXP3, and HELIOS when compared to TNFR2^+^ Tregs (**Supplemental Figures 7C, D**). Together, these results demonstrated a role of TNFR2 in promoting human Treg ex-vivo proliferation and a potential role in preserving Treg lineage by sustaining FOXP3, HELIOS, and CD25 expression.

Finally, we exposed TNFR2KO Tregs to IL-6 and TNF*a* to assess the requirement for TNFR2 in Treg proliferative boost by these cytokines shown in **Figure 1**. We observed that TNFR2KO Tregs had a diminished proliferative response to IL-6 and TNFα when compared to control Tregs (**Figure 4C**). Moreover, TNFR2KO Tregs had significantly decreased expression of key Treg lineage markers CD25, FOXP3, and HELIOS (**Figure 4D**). Together, these results suggest that TNFR2 signaling enhanced human Treg proliferation and safeguarded their identity during exposure to proinflammatory cytokines.

### Beadless Protocol for *Ex-Vivo* Expansion of Human Tregs

**Figure 5:**
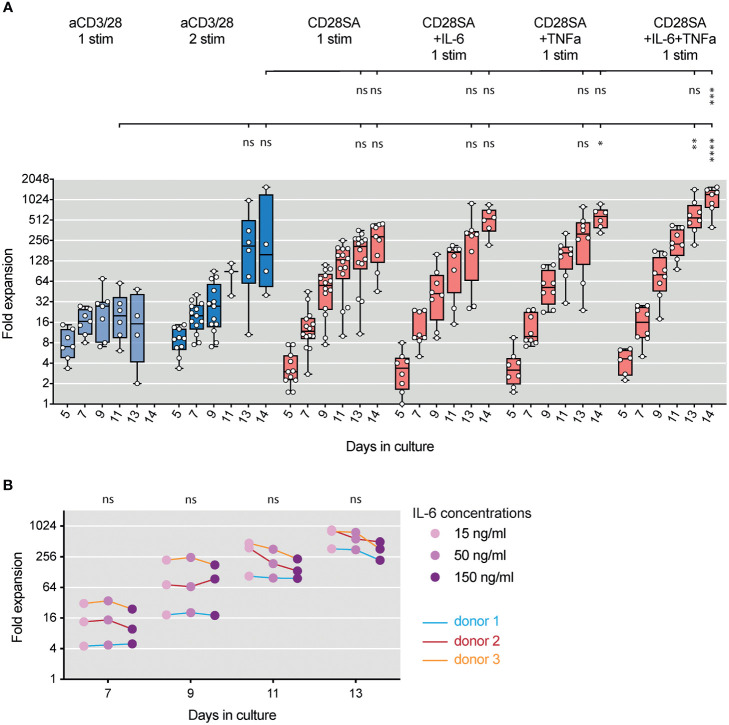
Beadless *ex-vivo* expansion of human Tregs. **(A)** FACS purified human Tregs were stimulated with either aCD3/28 beads or CD28SA and cultured in the presence of 300 IU/ml rhIL-2 with or without 150 ng/ml IL-6 and/or 50 ng/ml TNF*α* for up to 14 days. Expansion kinetics of Tregs from 7 to 14 unrelated donors in independent experiments are shown. Statistical significance was assessed using one-way ANOVA and Dunnett’s multiple comparisons posttest using aCD3/28 1 stim Day 13 and aCD3/28 2 stim Day 14 as reference. **(B)** FACS purified human Tregs were stimulated with CD28SA and cultured in the presence of 300 IU/mL rhIL-2, 50 ng/ml TNF*α* and varying concentrations of IL-6 (15 ng/ml, 50 ng/ml, and 150 ng/ml) as indicated. Expansion kinetics over 14 days of Tregs of from 3 unrelated donors in 3 independent experiments are shown. Data connected by the same line are from the same donor. Statistical significance was assessed using one-way ANOVA with Geisser-Greenhouse’s correction and Dunnett’s multiple comparisons posttest using 150 ng/ml IL-6 as reference. *p* values are marked as ns, not significant, *p < 0.05, **p < 0.01, ***p < 0.001, ****p < 0.0001.

*Ex-vivo* expanded human Tregs are currently being evaluated in clinical trials in transplantation and autoimmune diseases (58). Current Treg manufacturing processes rely on multiple rounds of stimulation with aCD3/28 beads (59). Our results of highly efficient expansion of stable human Tregs using one cycle of CD28SA stimulation in the presence of IL-2, IL-6, and TNF*a* prompted us to consider this protocol as an alternative approach to expand Tregs for clinical use. We thus compared rates of Treg expansion induced with 1 or 2 rounds of aCD3/28 bead stimulation versus those achieved with single round of CD28SA stimulation with or without the addition of IL-6 and TNF*a*. Tregs stimulated with aCD3/28 beads entered cell expansion more rapidly than that induced by CD28SA, but the cells rested by day 9 and required restimulation to proliferate again (**Figure 5A**). In contrast, Tregs stimulated with CD28SA continue to proliferate and began to rest by 14 days after stimulation. Addition of IL-6, TNF*a*, or both did not lead more rapid entry into cell division, but more persistent proliferation resulting in more total cell yields at the end of the two-week expansion (**Figure 5A**). Given the concern of the reported negative impact of IL-6 on Tregs, we determined if the persistent Treg proliferation can be achieved using less IL-6. Altering IL-6 concentrations from 15 ng/ml to 150 ng/ml in the context of the beadless protocol resulted in similar Treg expansion yields (**Figure 5B**).

The pattern of persistent proliferation after CD28SA stimulation in the presence of TNF*a* and IL-6 when compared with aCD3/28 bead stimulation suggested that Tregs cultured in these two protocols were in distinct metabolic states. We thus performed metabolomic profiling of aCD3/28 bead-stimulated versus CD28SA+IL-6+TNF*a* beadless-stimulated Tregs by using capillary electrophoresis mass spectrometry. Intracellular concentrations of 116 metabolites involved in glycolysis, pentose phosphate pathway, TCA cycle, lipid metabolism, urea cycle, and polyamine, creatine, purine, glutathione, nicotinamide, choline, and amino acid metabolisms were captured (**Supplemental Table 1**). We selected day 7 for the comparison because the Tregs in both protocols were briskly proliferating and had comparable fold expansion at that time, but about to diverge in the rate of proliferation.

**Figure 6:**
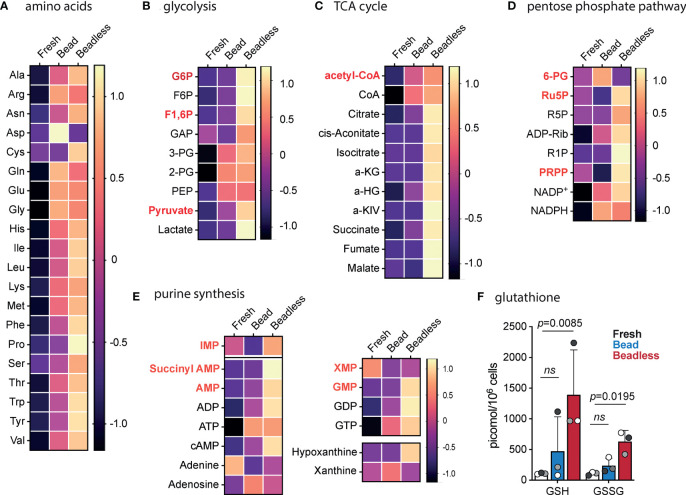
Metabolomic profile of Tregs before and after bead or beadless activation. Tregs were purified from 3 unrelated healthy donors. Tregs from each donor were divided into three parts: fresh Tregs without *in vitro* stimulation, Tregs expanded with aCD3/28 beads and IL-2 for 7 days (Bead), and Tregs expanded with CD28SA, IL-6, TNF*α*, and IL-2 for 7 days (Beadless). Intracellular metabolites were extracted and subjected to capillary electrophoresis mass spectrometry to profile 116 metabolites. The amount of each metabolite was normalized to the cell number and expressed as pmol/10^6^ cells. The data for each metabolite were then normalized to the mean of all the samples and log2 transformed so that differences among the three experimental conditions can be compared across different metabolites. Non-detected metabolites were given a value of 2^-52^. The transformed data for amino acids **(A)**, glycolysis **(B)**, TCA cycle **(C)**, pentose phosphate pathway **(D)**, and purine synthesis **(E)** are summarized as heatmaps. Products of rate-limiting steps or key metabolites are highlighted with bold red text. **(F)** Intracellular concentration of reduced (GSH) and oxidized (GSSG) glutathione are shown. Circles represent individual data and data from the same Tregs donor are represented by the same fill color. Statistical significance was determined using ratio paired t test. *p* values are as marked. ns, not significant, *p* > 0.05.

Activated Tregs with beads or the beadless protocol had significantly higher concentrations of all 20 amino acids than freshly purified Tregs (**Figure 6A**). All the amino acids, except aspartate, were present at equal or higher concentration in Tregs expanded with the beadless protocol when compared to those expanded with aCD3/28 beads. It was also notable that cysteine was only detected in Tregs stimulated with the beadless protocol and not detected in fresh or bead-stimulated Tregs. Both aspartate and cysteine are non-essential amino acids, and their altered levels may indicate altered metabolism of these amino acids in Tregs under different culture conditions.

Glycolysis was activated by both protocols with a trend of higher concentrations of glycolytic intermediates in the beadless-stimulated Tregs (**Figure 6B**). Notably, Tregs in the beadless protocol contained 10 times higher concentration of lactic acid compared to bead stimulated Tregs, indicating highly active glycolytic activities. Concurrently, beadless-stimulated Tregs had higher oxidative phosphorylation (OXPHOS) activities indicated by the higher concentrations of TCA intermediates when compared to bead stimulated Tregs, which were nearly depleted of TCA intermediates of cis-aconitate, isocitrate, alpha-ketoglutarate, fumarate and malate (**Figure 6C**). Thus, Tregs in the beadless protocol were in a high energy state with concurrent activation of both glycolysis and OXPHOS whereas bead stimulated Tregs mostly relied on glycolysis for energy production.

The pentose phosphate pathway also utilizes glycolysis intermediates for purine and pyrimidine synthesis. Intermediates in the pentose phosphate pathway were elevated in the beadless protocol expanded Tregs when compared to fresh and bead stimulated Tregs (**Figure 6D**). This correlated with higher concentrations of intermediates of purine biosynthesis (**Figure 6E**). The pentose phosphate pathway also converts NADP+ to NADPH, thus an important regulator of the redox state of the cell. Moreover, NADPH also supports *de novo* fatty acid synthesis. NADP+ and NADPH were present at comparable elevated concentrations in bead versus beadless stimulated Tregs (**Figure 6D**) whereas the NADPH to NADP+ ratios were both comparably reduced from the fresh Treg baseline (**Supplemental Table 1**), suggesting higher antioxidant demand and/or higher fatty acid synthesis in activated Tregs. NADPH protects mitochondria against oxidative stress by transferring its reductive power to oxidized glutathione disulfide (GSSG) to generate reduced glutathione (GSH). GSH and GSSG concentrations were consistently low in fresh Tregs, variably and moderately increased in bead stimulated Tregs, and significantly increased in Tregs activated with the beadless protocol (**Figure 6F**). Beadless protocol Tregs had higher levels of total glutathione, suggestive of greater buffering ability for reactive oxygen species (ROS). The increased energy production and *de novo* nucleotide synthesis combined with higher antioxidant potential may underscore the better metabolic fitness and more persistent proliferation of Tregs stimulated with the beadless protocol when compared to those stimulated with aCD3/28 beads.

### TNFα and IL-6-Exposed Tregs Maintained Their Function *In Vitro* and *In Vivo*

**Figure 7:**
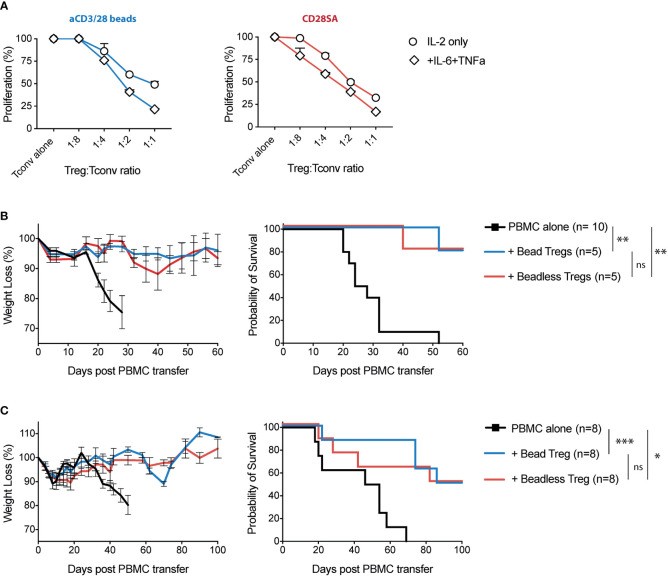
Tregs expanded with the beadless protocol maintained their *in-vitro* and *in-vivo* suppressive function. **(A)** FACS purified human Tregs were stimulated with either aCD3/28 beads or CD28SA and cultured in the presence of 300 IU/ml rhIL-2 with or without TNF*α* and/or IL-6 for 9 days, as indicated. In addition, FACS purified human Tconvs were stimulated with aCD3/28 beads and cultured in parallel to autologous Tregs. At day 7 of culture, expanded Tregs were harvested and then co-cultured with 5x 10^4^ ex-vivo expanded Tconv at Treg/Tconv ratios of 1:1, 1:2, 1:4 and 1:8 in the presence of aCD3/CD28 beads (1:10 bead to Tconv ratio). Proliferation was measured using ^3^[H]-thymidine incorporation in triplicate wells on day 4 of co-culture. Results shown are representative of 2 independent experiments using 2 unrelated donors. **(B)** At day 9 of culture, either standard protocol (aCD3/28 beads and IL-2) or beadless protocol (CD28SA, IL-2, IL-6 and TNF*α*) expanded Tregs were adoptively co-transferred into sub lethally irradiated NSG mice along with autologous PBMC. Results shown are from 1 experiment with a total of at least 5 mice per group. **(C)** Same as panel **(B)**, except the Tregs were injected 4 days after the PBMC infusion. Weight loss and survival were monitored every other day. Results shown are summary of 2 independent experiments using 2 unrelated donors with a total of 8 mice per group. Kaplan-Meier survival graphs are shown, and a log-rank comparison of the groups was used to calculate *p* values. *p* values are marked as ns, not significant, *p* > 0.05, *p < 0.05, **p < 0.01, ***p < 0.001.

Lastly, we compared the function of Tregs expanded using aCD3/28 beads versus the beadless protocol. For the *in vitro* suppression assay, we used aCD3/28 beads expanded CD4^+^CD25^-^CD127^hi^ Tconv as responders and mixed in titrated numbers of Tregs expanded with either aCD3/28 beads or CD28SA, with or without IL-6 and TNF*a*. Tregs expanded with various protocols maintained high suppressive capacity, suggesting that *ex-vivo* exposure to IL-6 and TNF*a* did not negatively affect Treg suppressive function (**Figure 7A**).

To measure the *in-vivo* suppressive function of Tregs, we used models of xenogeneic GVHD in the NSG mice. Intravenous injection of 5 x 10^6^ PBMC’s into sub lethally irradiated NSG mice resulted in the development of GVHD that was lethal in 20 to 30 days (**Figure 7B**). Co-transfer of 5 x 10^6^ Tregs expanded with standard aCD3/28 bead protocol or the beadless protocol significantly attenuated weight loss and improved survival from GVHD in most of the mice (**Figure 7B**). To compare the function of the Tregs expanded with the standard versus beadless protocols in a more challenging condition, we infused Treg 4 days after PBMC injection. It has been previously reported that co-infusion of Treg with GVHD-inducing PBMCs effectively prevents Tconv proliferation and improves survival, while delayed Treg infusion requires higher doses of Tregs to reverse disease that has already been initiated (60). Indeed, an increased percentage of humanized NSG mice treated with late Treg infusion eventually succumbed to GVHD compared to the GVHD prevention model with PBMC and Treg co-infusions. However, no differences were identified between Tregs expanded with the standard protocol versus the beadless protocol in their ability to prevent weight loss and reduce mortality (**Figure 7C**).

To assess the phenotypic stability of Tregs after infusion, we injected 2x10^6^ autologous PBMCs into sub lethally irradiated NSG mice 9 days prior to adoptively transferring CFSE-labeled Tregs expanded with either the standard bead-based or the beadless protocol. We collected blood and lung tissues 5 days after Treg infusion to analyze the Treg expression of FOXP3. Beadless expanded Tregs maintained their FOXP3 expression when compared to the CD8^+^ T cells among the PBMCs which were largely FOXP3^-^ (**Supplemental Figure 8A**). We observed slightly lower levels of FOXP3 expression in beadless protocol expanded Tregs when compared to bead-expanded Tregs (**Supplemental Figures 8B, C**) and this was associated with significantly more proliferation of the beadless Tregs (**Supplemental Figure 8D**). In summary, these results demonstrated that ex-vivo expanded Tregs exposed to IL-6 and TNF*a* maintained their function *in vitro* and *in vivo* in humanized mouse models of GVHD.

## Discussion

In this study, we challenged purified human peripheral blood Tregs ex-vivo with IL-6 and TNF*a* to determine their direct effect on Tregs. Our data collectively demonstrate that human Tregs respond to these cytokines positively by increasing their proliferation while preserving their lineage identity.

A Treg-enhancing role of TNF*a* and TNFR2 has been reported in various mouse models and with human Tregs *ex vivo*. In this study, we have identified 3 effects of TNF*a* on human Tregs. First, by using TNFR blockers and CRISPR/Cas 9 mediated *TNFR2* gene deletion, we further show that activated human Tregs engage TNF*a*-TNFR2 in an autocrine or paracrine fashion for persistent proliferation. Exogenous provision of TNF*a* and IL-6 further boosted this effect, suggesting the endogenous production of TNF*a* and LT*a* does not maximally activate this pathway. Second, TNF*a* helps to preserve Treg lineage identity by sustaining high expression of FOXP3 and HELIOS. This finding is consistent with a previous report that TNFR2 signaling in human Tregs induces EZH2 to preserve Treg lineage identity (61). Lastly, TNF*a* increases Treg secretion of CCL3, CCL4, and CCL5. It has been shown previously that mouse Tregs produce CCL3 and CCL4 to attract activated T cells to suppress them (62). Together, these findings point to multiple mechanisms of TNF*a*-TNFR2 in promoting Treg numerical dominance lineage stability, and function at sites of inflammation.

In contrast to TNF*a*, IL-6 has mostly been thought to subvert Treg functions. Our finding of a lack of destabilizing effect of IL-6 on human Tregs despite the use of supraphysiological concentration of IL-6 and reduced IL-2 is thus unexpected. Further review of the literature shows a unanimous conclusion that IL-6 antagonizes *in vitro* induction of Tregs from Tconvs by driving the cells to differentiation into Th17 effectors (15). Moreover, there is consensus that IL-6 enhances Teff activation to resist suppression by Tregs (13, 16). The body of literature on a direct role of IL-6 on lineage committed Tregs, especially on human Tregs, is limited and more controversial. While it is difficult to pinpoint the cause of divergent findings between this study and previous studies that reported Treg destabilizing effects of IL-6, we suspect that differences in experimental setup may have contributed. An uncertainty when working with human Tregs is the identity of the cells at the start of the experiments depending on the markers used for Treg isolation. In this study, we isolated human Tregs from peripheral blood using FACS with markers of CD4^+^CD25^+^CD127^lo/-^. The addition of CD127 allows isolation of human Tregs with higher purity and higher yield (63, 64). Isolation of human Tregs based on the expression of CD4 and CD25 using magnetic activated cell sorting often results in less pure Tregs and outgrowth of Tconvs that may be mistaken for destabilized Tregs. Our findings with Tregs from many unrelated donors over the span of 4 years show that acute high dose exposure to IL-6 does not cause destabilization of human Tregs.

In this study, we noted significantly more robust proliferation of Tregs stimulated with CD28SA when compared to aCD3/28 bead stimulation. Furthermore, IL-6 and TNF*a* synergistically enhanced the proliferation stimulated by CD28SA. It has been reported that IL-6 promotes TNFR2 expression by Tregs in malignant ascites (65), which may explain the synergy we have observed. It is worth noting that aCD3/28 bead stimulated faster entry into cell cycle but earlier rest akin to logarithmic growth; whereas CD28SA stimulated Tregs showed slower start but more robust and persistent cell cycling as seen in exponential growth, especially when IL-6 and TNF*α* are added. This suggests that Tregs are capable of exponential growth and the pattern of expansion stimulated by aCD3/28 beads may be a result of negative feedback that limits proliferation. The biochemical basis of the distinct proliferation patterns remains to be elucidated. In Tconvs, TCR stimulation in the absence of CD28 signaling activate nuclear factor of activated T-cells that is not balanced with concurrent activation of NF-κB and activator protein 1, which leads induction of E3 ubiquitin ligases Cbl-b and cell cycle arrest (66). CD28-mediated costimulation complement TCR signaling by activating NF-κB and AP1 while releasing the cells from the inhibitory effect of Cbl-b (66). We speculate that stimulation of Tregs with strong TCR agonist such as bead-bound aCD3/28 induces imbalanced activation of transcription factors that triggers negative feedback to limit Treg proliferation, resulting in short bursts of proliferation followed by stagnation (67). On the other hand, CD28SA stimulated T cell activation depends on the expression of TCR but does not ostensibly induce activation of signaling intermediates immediately downstream of the TCR such as CD3 chains and ZAP70 (68, 69). Thus, CD28SA delivers a strong costimulatory signal in the context of a weak TCR signal (70–72), which may avoid the engagement of negative feedback loops that leads to earlier cell cycle arrest. The addition of IL-6 and TNF*a* may further engage complementary signaling pathways to boost Treg proliferation. Ongoing work in our laboratory is actively testing these predications.

Another non-mutually exclusive explanation for the distinct Treg proliferation patterns is the divergent metabolic programs induced by aCD3/28 bead versus CD28SA plus IL-6 and TNF*a* (beadless protocol). Tregs undergo metabolic reprogramming after activation to accommodate the anabolic and energy demands for cell proliferation (73). Previous studies have reported either preferential fatty acid oxidation (FAO)-fueled oxidative phosphorylation over glycolysis by Tregs (74–76) or reliance on both glycolysis and FAO to support Treg proliferation and suppressive function (77, 78). In our metabolomic analysis, both bead and beadless stimulations led to increased intracellular amino acid concentrations, energy production, and nucleotide synthesis when compared to freshly isolated Tregs, consistent with their anabolic state. We noted three major distinctions in the metabolic state of Tregs stimulated with aCD3/28 beads versus the beadless protocol. First, while both protocols increased glycolytic flux, the beadless protocol Tregs had a slightly higher concentration of pyruvate, but 10 times more intracellular lactate. The conversion of pyruvate to lactate is coupled with oxidation of NADH to replenish the NAD+ pool in the cytosol, which is essential in preventing stagnation of glycolysis from NAD+ shortage (79). This suggests that Tregs in the beadless protocol had more active glycolysis. Second, the beadless protocol stimulated Tregs had higher concentrations of all TCA intermediates measured, whereas intermediates downstream of citrate were almost completely depleted in aCD3/28 bead stimulated Tregs. This suggests that the aCD3/28 bead stimulated Tregs have very low level of OXPHOS and relied mostly on glycolysis for energy production. Third, concentrations of total and reduced glutathione were consistently higher in the beadless protocol stimulated Tregs, suggestive of greater buffering capability for ROS. Intense OXPHOS can increase the generation of ROS leading to cell death. Therefore, reduced glutathione and other antioxidants are essential for maintaining cells in a high energetic state by scavenging ROS (80). GSH deficiency in Tregs results in an imbalanced intracellular redox state and impaired suppressive function (81). Overall, our data indicate that the beadless protocol stimulated Tregs have better metabolic fitness that may contribute to their persistent proliferation.

It is unclear at this point of our investigations which component of the beadless protocol contributed to the more favorable metabolic program in Tregs. CD28 costimulation is required for inducing glycolysis in activated T cells during proliferation (82, 83), and can also prime mitochondria for increased OXPHOS demand (84). CD28SA has been shown to drive T effector memory cells into an adaptable metabolic state that can flexibly maximize glycolysis and OXPHOS potentials depending on glucose and oxygen availability (85), but the impact of CD28SA on metabolic programming of Tregs remains uncertain. Another component of the beadless protocol, TNF*a*, may act through TNFR2, to induce a glycolytic switch in Tregs coupled with shunting of intermediates into the TCA cycle, thereby promoting anabolic biosynthetic processes (86). Lastly, it has been reported that STAT3 can localize in the inner membrane of mitochondria and enhance the efficiency of electron transport chain and reduce the generation of ROS (87). Further research is needed to dissect the role of the individual components of the beadless protocol on Treg metabolism, which is currently ongoing in our laboratory.

A practical implication of our data is a new protocol for ex-vivo human Treg expansion. One of the challenges facing Treg therapy is the ability to reliably manufacture enough Tregs without the need for repeated stimulations that negatively affect Treg stability (88–90). The beadless protocol promotes highly efficient Treg expansion with only one cycle of CD28SA stimulation. Another advantage of the beadless protocol is the use of all soluble reagents, thus harvesting of Treg products at the end of the expansion is simplified. Thus, the beadless protocol offers several improvements in the Treg manufacturing process.

Taken together, our findings show that human Tregs positively respond to TNF*a* and IL-6 by increased proliferation while safeguarding their lineage stability. With proper stimulation and the right cytokine milieu, human Tregs can grow exponentially, which may be a result of balanced transcription and metabolic programing. We speculate that increased proliferation in response to inflammatory cytokines allows Tregs to scale to inflammation to restore immune homeostasis. These properties of Tregs may be harnessed to improve the manufacturing of therapeutic Tregs for autoimmune diseases and transplantation.

## Data Availability Statement

The original contributions presented in the study are included in the article/**Supplementary Material**. Further inquiries can be directed to the corresponding author.

## Ethics Statement

The animal study was reviewed and approved by UCSF IACUC protocol No. AN183959-02.

## Author Contributions

Designed the project: QT, NS. Supervised the project: QT, FV. Designed experiments: NS, QT. Performed experiments: NS, YP, LF, VN, ER. Analyzed data: NS, YP, QT. Provided reagents and advice: YDM, FV. Wrote the manuscript: NS, YP, QT. All authors contributed to the article and approved the submitted version.

## Funding

This work was supported by grants from NIAID (Clinical Trials in Organ Transplant, 0255-B011-4609 and 0255-B012-4609, which were ancillary to a NIAID grant U01 AI113362), The Leona M. and Harry B. Helmsley Charitable Trust (2018PG-T1D042), NIDDK (UC4 DK116264). This works used the UCSF Flow Cytometry Core, which is supported in part by the Diabetes Research Center Grant from NIDDK (P30 DK063720 and P30 DK063720). NS was supported by a fellowship grant from the American Society of Nephrology. YDM was supported by the Swiss National Science Foundation (Mobility Grant no. P300PB_174500) and a fellowship grant from the University Hospital of Geneva. LF was the Jeffrey G. Klein Family Diabetes Fellow.

## Conflict of Interest

QT is a co-founder and scientific advisor of Sonoma Biotherapeutics. QT, NS, and FV are co-inventors of a patent on manufacturing Tregs based on results from this work.

The remaining authors declare that the research was conducted in the absence of any commercial or financial relationships that could be construed as a potential conflict of interest.

## Publisher’s Note

All claims expressed in this article are solely those of the authors and do not necessarily represent those of their affiliated organizations, or those of the publisher, the editors and the reviewers. Any product that may be evaluated in this article, or claim that may be made by its manufacturer, is not guaranteed or endorsed by the publisher.
