# Supplementary material for: IL-6 and TNFα Drive Extensive Proliferation of Human Tregs Without Compromising Their Lineage Stability or Function
Source: Front Immunol. 2021 Dec 23;12:783282. doi: 10.3389/fimmu.2021.783282 (PMC8732758; doi:10.3389/fimmu.2021.783282)
Supplement: Supplementary file 1 [file DataSheet_1.pdf]

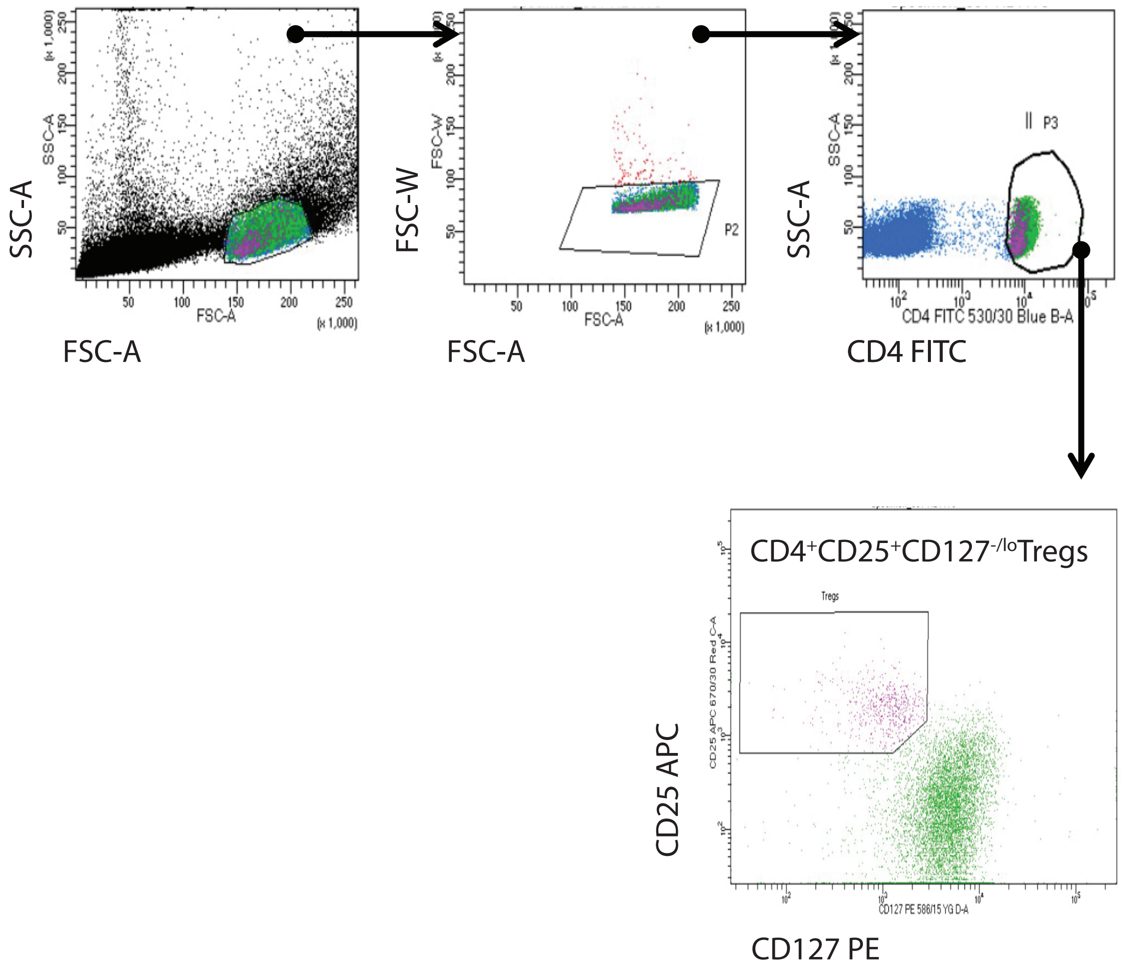


**Supplemental Figure 1. An example of FACS gating strategy for Treg purification.**

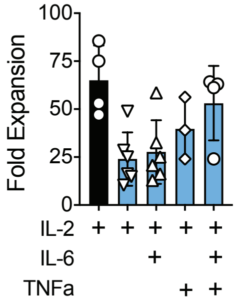


**Supplemental Figure 2. Tconv versus Treg proliferation after stimulation with aCD3/28 beads.** FACS purified $CD4^{+}CD25^{-}CD127^{+}$ Tconv (black) and $CD4^{+}CD25^{+}CD127^{-/lo}$ Tregs (blue) were stimulated with aCD3/28 beads and cultured for 12 days in medium supplemented with IL-2 (300IU/mL). IL-6 and TNFa were added in some Treg cultures as indicated. Results shown are a summary of 3-6 experiments using cells from unrelated normal donors in each experiment. Each symbol represents one culture with a distinct donor.

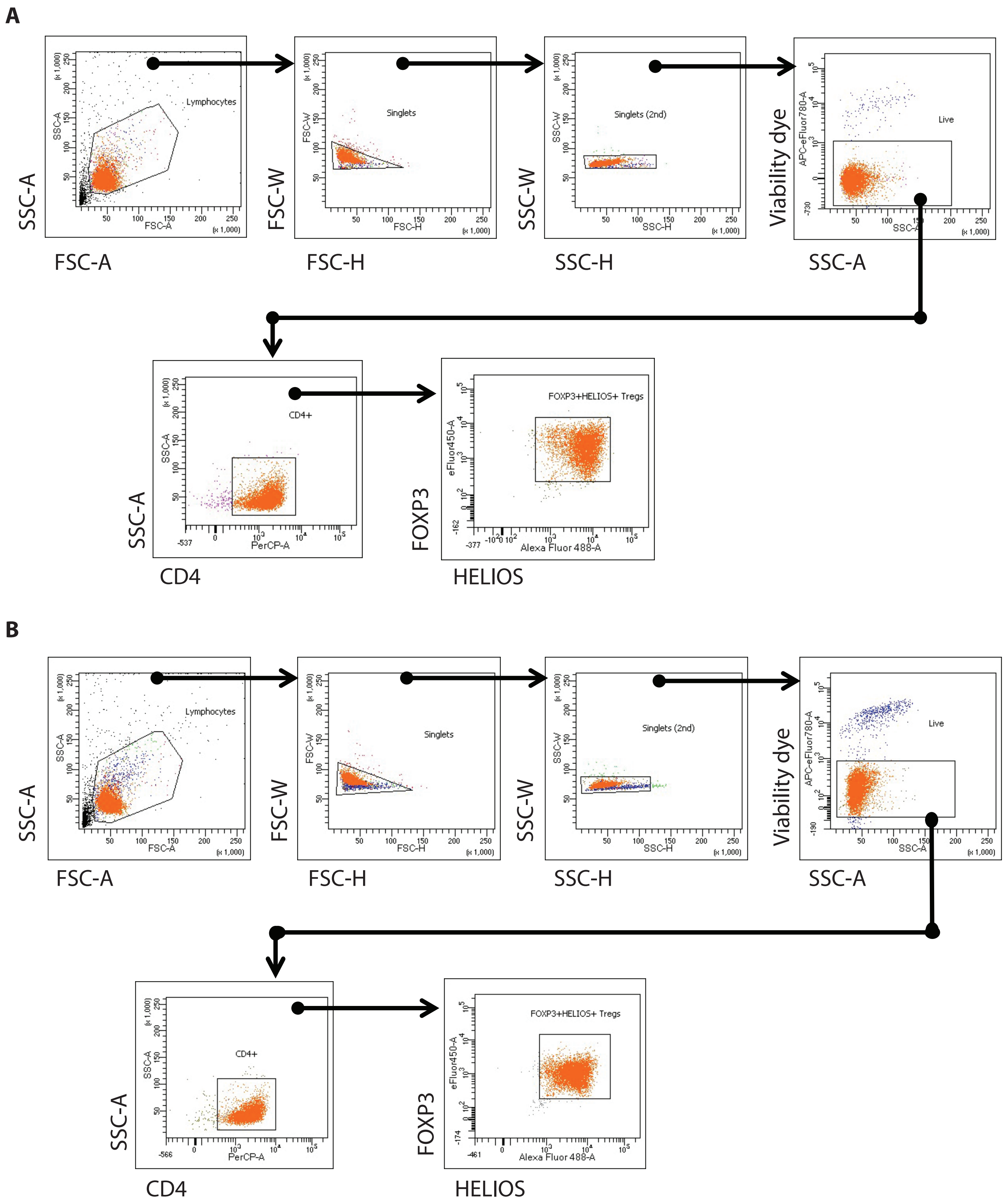


**Supplemental Figure 3.** An example of flow cytometric analysis gating strategy of a Treg culture on day 9 (A) and day 14 (B). Tregs were stimulated with aCD3/28 beads and supplemented with 300IU/mL IL-2.

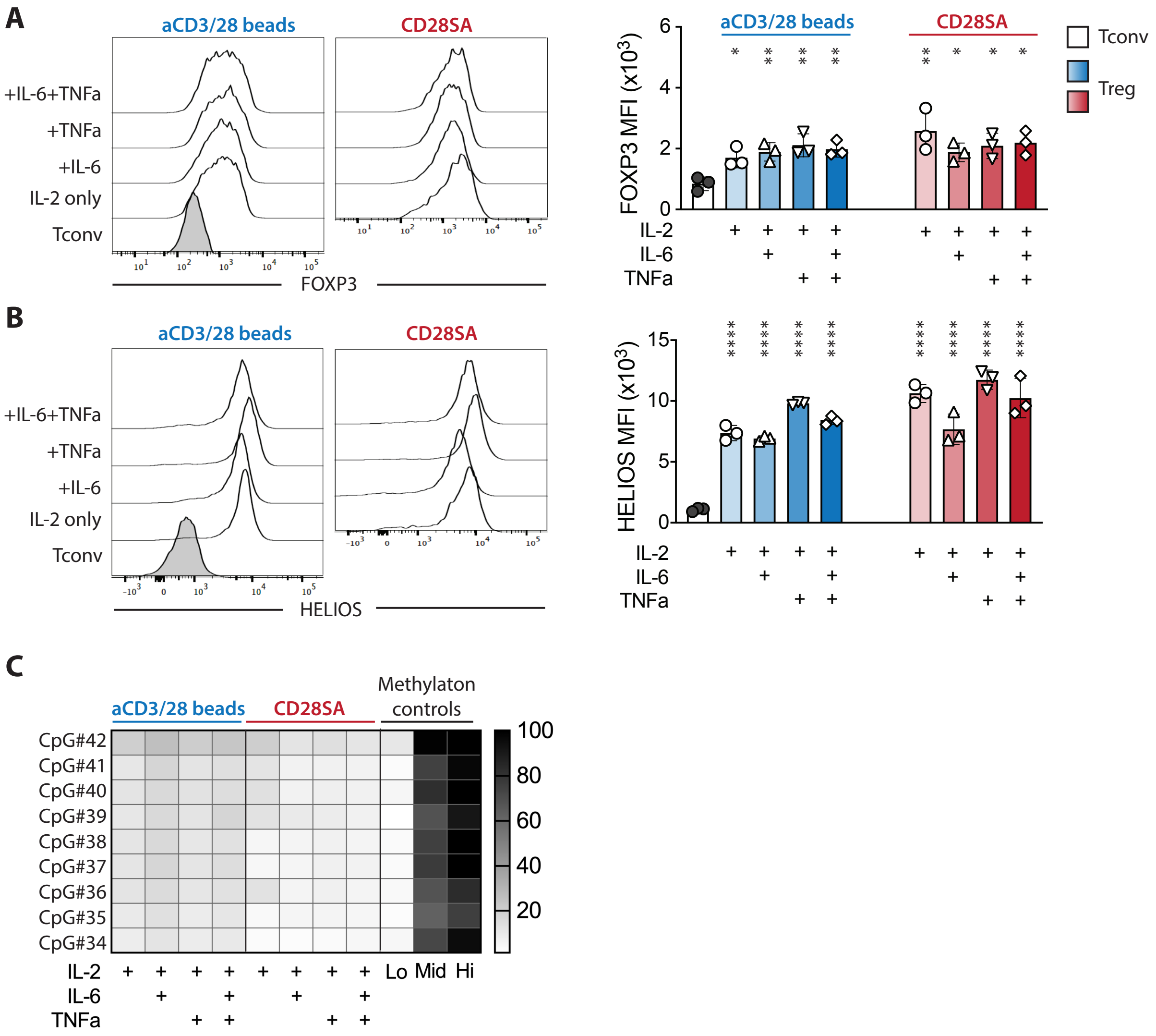


**Supplemental Figure 4. Tregs exposed to IL-6 and/or TNFa maintained their lineage identity despite decreased IL-2.** FACS purified human Tregs were stimulated with either aCD3/28 beads or CD28SA and cultured in the presence of 15 IU/ml rhIL-2 with or without TNFa and IL-6 as indicated. (**A** and **B**) Flow cytometric analysis of FOXP3 (A) and HELIOS (B) expression in Tregs on day 9 after stimulation. Representative histograms (left) and summaries of MFI's from 3 independent experiments (right) are shown. Statistical significance of differences was assessed using one-way ANOVA and Dunnett's multiple comparisons test using Tconv (Panels A and B) as a baseline reference. p values are marked as *=p<0.05, **=p<0.01, ****=p<0.0001. (**C**) Heatmap summary of TSDR demethylation of Tregs expanded in various conditions. Results shown are averages of Treg cultures using 2 unrelated male donors in 2 independent experiments.

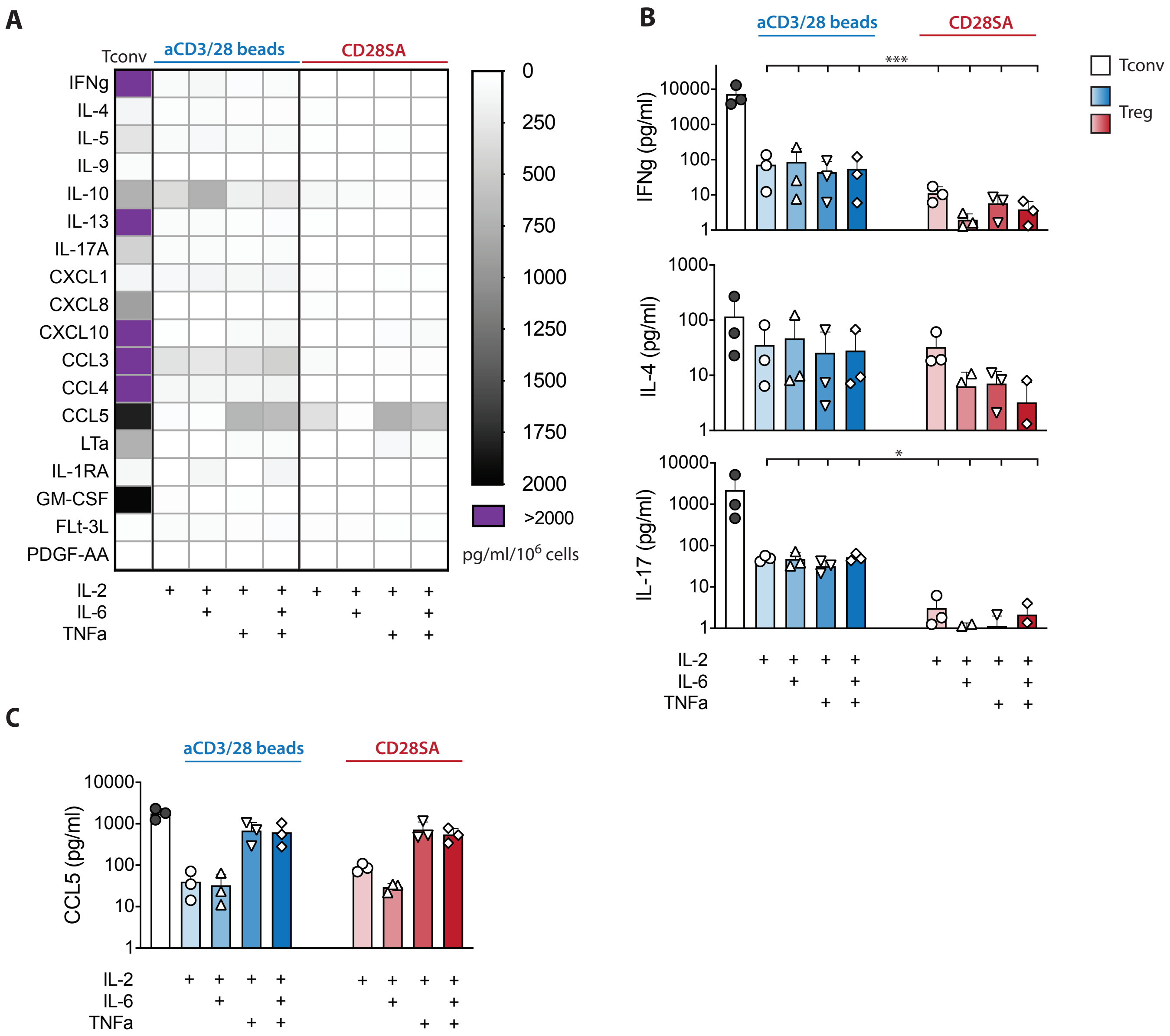


**Supplemental Figure 5. TNFa and IL-6 expanded Tregs did not secrete proinflammatory cytokines despite decreased IL-2 challenge.** FACS purified human Tregs were stimulated with either aCD3/28 beads or CD28SA and cultured in the presence of 15 IU/ml rhIL-2 with or without TNFa and IL-6 as indicated. Cytokine and chemokine secretion in the culture supernatant of various Treg cultures was assessed using a multiplex Luminex panel. Supernatant in aCD3/28 bead stimulated Tconv cultures are included as a reference. (**A**) Heatmap summary of cytokines and chemokines that were present in any of the culture condition is shown. (**B**) IFN-g, IL-4, and IL-17 concentrations in the Day 7 culture supernatants are shown. (**C**) CCL5 and LTa concentrations in the Day 7 culture supernatant are shown. Results shown are summaries of 3 independent experiments using cells from 3 unrelated donors. Statistical significance of differences was assessed using one-way ANOVA and Dunnett's multiple comparisons posttest using Tconv (Panels B and C) as a baseline reference. p values are marked as *=p<0.05, ***=p<0.001.

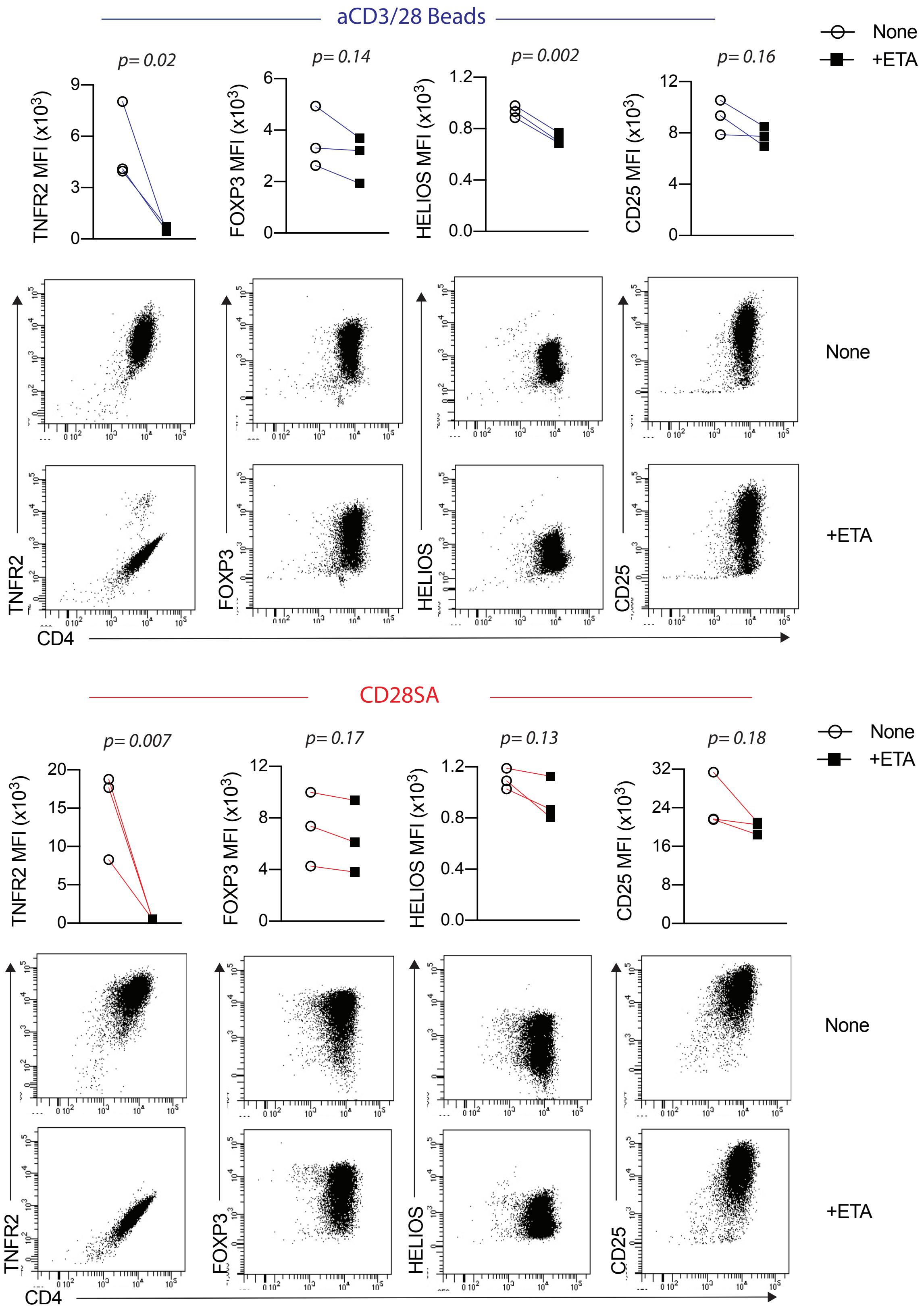


**Supplemental Figure 6. Etanercept-treated Treg expression of TNFR2, FOXP3, HELIOS, and CD25.** Summary of MFI of TNFR2, FOXP3, HELIOS, and CD25 of Tregs cultured in the presence or absence of etanercept, assessed using flow cytometry on day 8 of culture. Representative flow plots are shown below the summary graphs. Paired t-test was used to determine statistical significance of the differences and *p* values are stated above the graphs. Results shown are a summary of 3 independent experiments using cells from 3 unrelated donors.

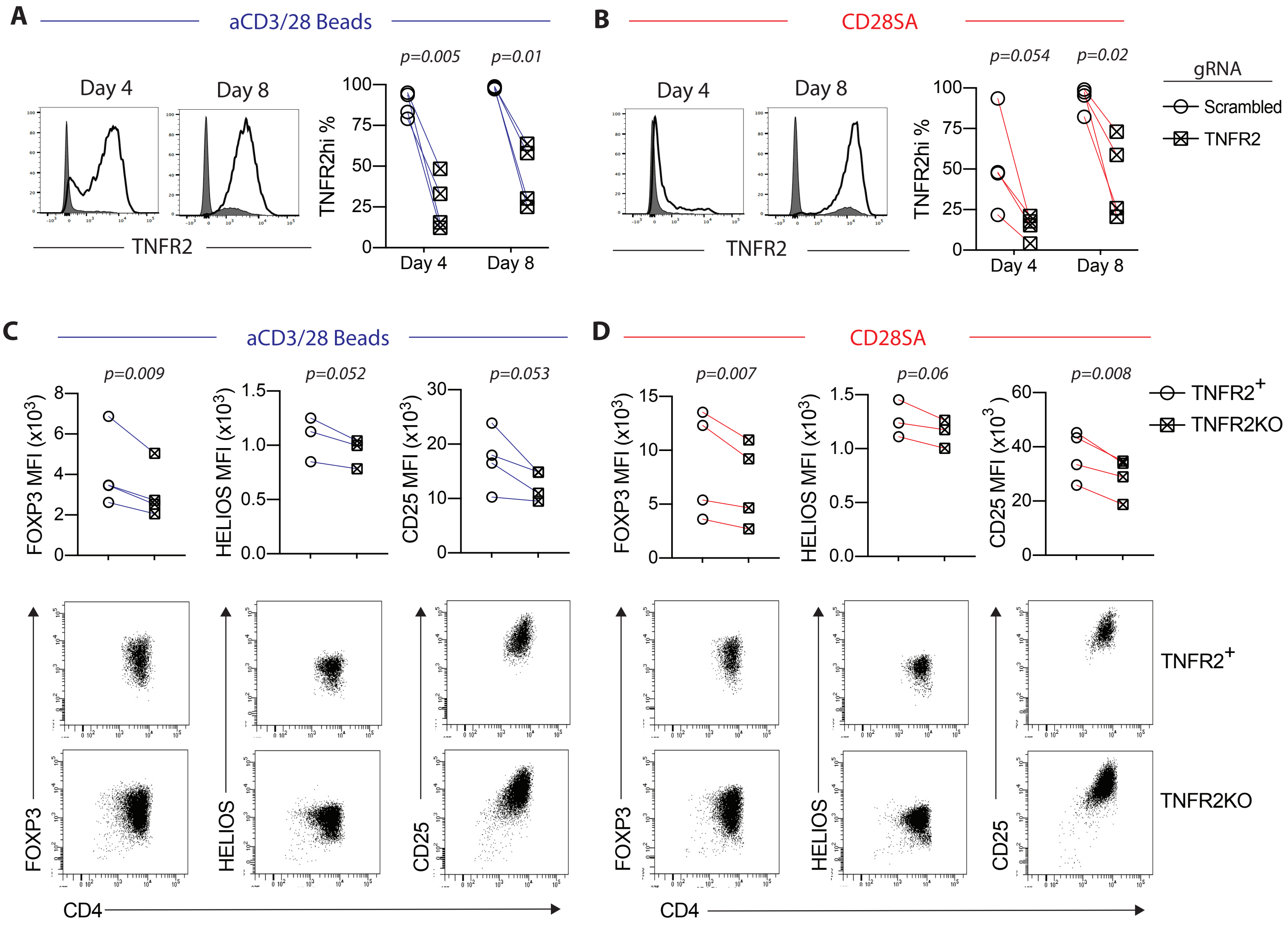


**Supplemental Figure 7. TNFR2KO Treg expression of FOXP3, HELIOS and CD25.** Human Tregs were gene edited using the CRISPR-Cas9 technology to delete the *TNFR2* gene prior to stimulation with either aCD3/CD28 beads or CD28SA. TNFR2 expression on either (**A**) aCD3/28 bead or (**B**) CD28SA stimulated Tregs that were electroporated with scrambled gRNA (open histograms) or gRNA targeting the *TNFR2* gene (shaded histograms) was assessed using flow cytometry on day 4 and 8 of ex-vivo Treg culture. MFI's of FOXP3, HELIOS, and CD25 of either (**C**) aCD3/28 bead or (**D**) CD28SA stimulated TNFR2$^+$ versus TNFR2KO Tregs were assessed using flow cytometry on day 8 of culture. Representative flow plots are shown below the summary graphs. Paired t-test was used to assess statistical significance of the differences and *p* values are stated above the graphs. Results shown are a summary of 4 independent experiments using cells from 4 unrelated donors.

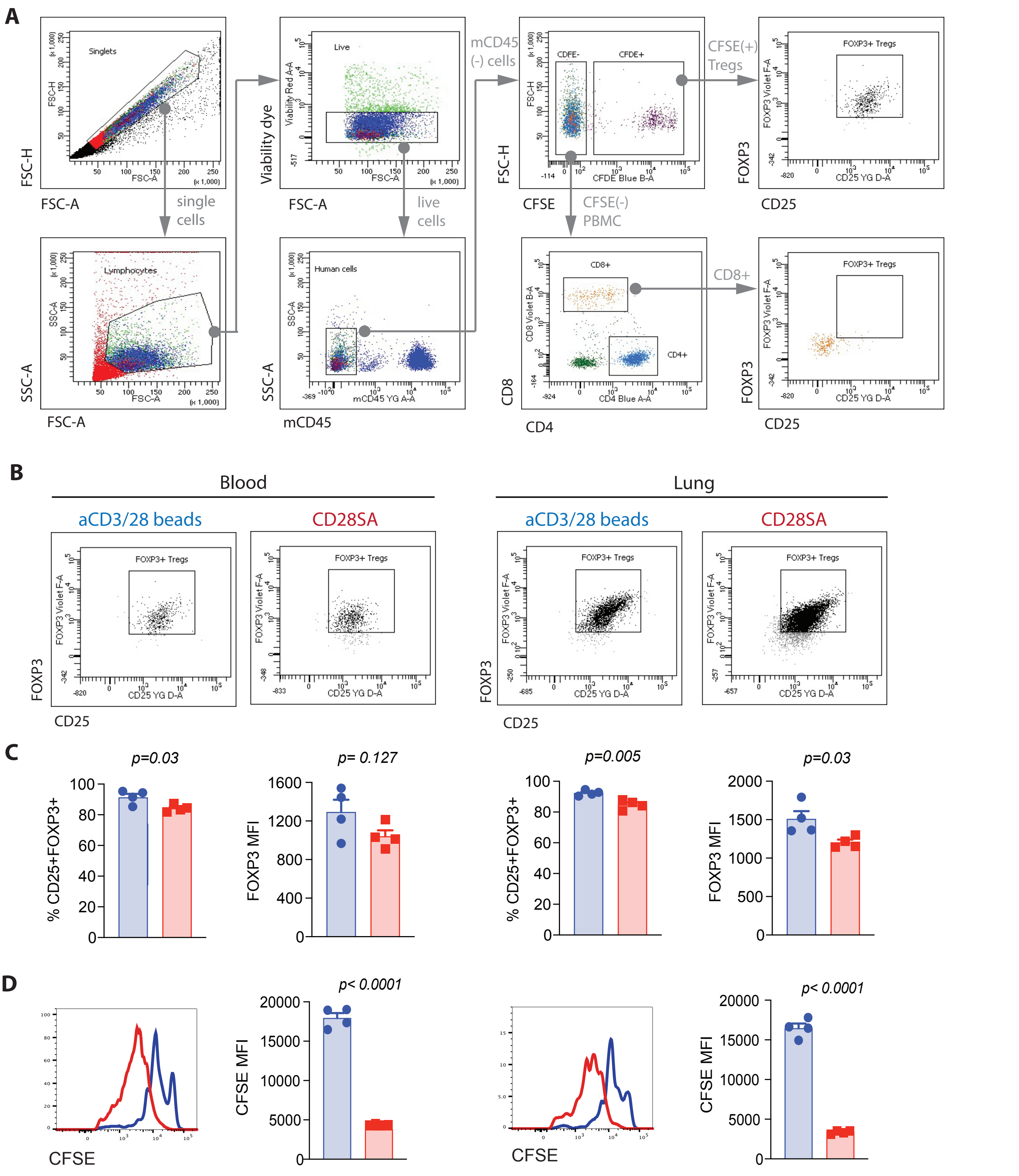


**Supplemental Figure 8. Analysis of bead- vs. beadless-expanded Tregs after infusion in NSG mice.** Irradiated NSG mice were infused with 2 million PBMC followed by an infusion of 4 million CFSE-labled bead-expanded or beadless-expanded Tregs 9 days later. On day 5 after Treg infusion, blood and lung tissue were collected for analyses. (**A**) Gating strategy to identify CFSE-labeled Tregs. CD8+ T cells among the PBMC were used as a reference for the CD25 vs. FOXP3 analysis gate. (**B**) Representative CD25 vs. FOXP3 flow plots of CFSE-labeled Tregs in peripheral blood and lung tissue. (**C**) Summary of %CD25 +FOXP3+ (left) and FOXP3 MFI (right) of 4 mice per group. (**D**) Representative CFSE histogram overlays of Tregs (left) and summary of CFSE MFI (right) of 4 mice per group. Results shown are summary of 1 experiment with 4 mice per group. Statistical significance was determined using unpaired t test and p values are stated above the graphs. mCD45: mouse CD45.
