# Supplementary material for: IL-6 and TNFα Drive Extensive Proliferation of Human Tregs Without Compromising Their Lineage Stability or Function
Source: Front Immunol. 2021 Dec 23;12:783282. doi: 10.3389/fimmu.2021.783282 (PMC8732758; doi:10.3389/fimmu.2021.783282)
Supplement: Supplementary file 2 [file Table_1.pdf]

Absolute Concentration of 116 Compounds (pmol/10^6 cells)

	Compound name	Pathway Label	PubChem CID	Fresh			Bead			Beadless		
				Donor 1	Donor 2	Donor 3	Donor 1	Donor 2	Donor 3	Donor 1	Donor 2	Donor 3
A_0001	NAD^+	NAD+	5893	7.1	6.7	6.4	160	141	215	421	227	327
A_0002	cAMP	cAMP	6076	0.2	0.3	0.3	0.5	0.5	0.4	1.4	0.7	0.7
A_0003	cGMP	cGMP	24316	N.D.	N.D.	N.D.	N.D.	N.D.	N.D.	N.D.	N.D.	N.D.
A_0004	NADH	NADH	439153	8.3	5.0	3.9	14	20	14	38	20	32
A_0005	Xanthine	Xanthine	1188	1.1	N.D.	0.4	1.6	N.D.	0.6	0.5	N.D.	0.8
A_0006	ADP-ribose	ADP-Rib	445794	0.05	0.04	0.03	0.6	0.4	1.0	1.5	0.6	1.5
A_0007	Mevalonic acid	Mevalonic acid	134965	N.D.	N.D.	N.D.	N.D.	N.D.	N.D.	N.D.	N.D.	N.D.
A_0008	UDP-glucose	UDP-Glc	8629	4.7	7.7	8.1	71	52	98	273	152	255
A_0009	Uric acid	Uric acid	1175	3.3	6.2	1.4	1.6	1.1	N.D.	2.1	0.3	N.D.
A_0010	$NADP^+$	NADP+	5886	2.0	0.9	0.8	3.1	4.1	4.4	5.9	4.3	5.3
A_0011	IMP	IMP	8582	77	10	7.4	2.1	2.8	16	83	23	51
A_0012	Sedoheptulose 7-phosphate	S7P	165007	N.D.	N.D.	N.D.	N.D.	N.D.	N.D.	N.D.	N.D.	N.D.
A_0013	Glucose 6-phosphate	G6P	5958	3.8	8.1	4.3	8.6	7.9	2.9	13	14	17
A_0014	Fructose 6-phosphate	F6P	603	1.4	2.8	1.5	3.5	3.0	1.8	5.3	6.3	7.6
A_0015	Fructose 1-phosphate	D-F1P	439394	N.D.	N.D.	N.D.	N.D.	N.D.	N.D.	14	7.9	N.D.
A_0016	Galactose 1-phosphate	Gal1P	123912	N.D.	N.D.	N.D.	N.D.	N.D.	N.D.	1.4	1.0	3.1
A_0017	Glucose 1-phosphate	G1P	65533	1.7	1.2	1.0	3.2	3.7	1.8	3.2	2.4	4.9
A_0018	Acetoacetyl CoA	AAcCoA	92153	N.D.	N.D.	N.D.	N.D.	N.D.	N.D.	N.D.	N.D.	N.D.
A_0019	Acetyl CoA	AcCoA	444493	0.2	0.2	0.13	1.0	1.3	1.6	2.0	0.09	3.6
A_0020	Folic acid	Folic acid	6037	0.3	0.3	0.3	1.2	1.5	0.7	0.4	0.2	0.6
A_0021	Ribose 5-phosphate	R5P	439167	3.3	3.8	2.1	1.9	2.7	4.2	5.6	1.8	6.7
A_0022	CoA	CoA	87642	0.8	0.6	0.4	1.7	1.8	3.2	2.6	2.4	2.9
A_0023	Ribose 1-phosphate	R1P	439236	1.4	0.9	0.9	2.1	N.D.	1.6	7.0	3.4	4.8
A_0024	Ribulose 5-phosphate	Ru5P	439184	0.4	0.8	N.D.	0.09	N.D.	0.3	1.0	0.5	1.4
A_0025	Xylulose 5-phosphate	X5P	439190	N.D.	N.D.	N.D.	N.D.	N.D.	N.D.	N.D.	N.D.	N.D.
A_0026	Erythrose 4-phosphate	E4P	122357	N.D.	N.D.	N.D.	N.D.	N.D.	N.D.	N.D.	N.D.	N.D.
A_0027	HMG CoA	HMG-CoA	445127	0.3	0.2	0.2	0.5	0.8	0.4	1.5	0.7	1.3
A_0028	Glyceraldehyde 3-phosphate	Glyceraldehyde 3-phosphate	729	0.8	1.2	0.9	N.D.	N.D.	1.6	1.6	0.2	4.2
A_0029	NADPH	NADPH	5884	6.5	4.0	3.3	10	14	8.2	12	7.8	11
A_0030	Malonyl CoA	Malonyl-CoA	644066	N.D.	N.D.	N.D.	0.4	0.7	0.2	0.3	0.12	0.4
A_0031	Phosphocreatine	Phosphocreatine	9548602	0.011	0.3	0.3	3.0	N.D.	0.05	2.8	0.12	N.D.
A_0032	XMP	XMP	73323	4.2	0.2	0.14	0.3	0.3	0.2	0.9	0.3	0.6
A_0033	Dihydroxyacetone phosphate	DHAP	668	3.9	3.1	0.7	N.D.	N.D.	1.8	1.4	2.0	4.6
A_0034	Adenylosuccinic acid	Succinyl AMP	447145	1.7	0.7	0.4	1.0	1.5	1.2	6.5	3.3	5.7
A_0035	Fructose 1,6-diphosphate	F1,6P	172313	7.3	30	18	27	26	18	43	59	34
A_0036	6-Phosphogluconic acid	6-PG	91493	1.5	11	7.9	21	9.7	4.8	6.7	3.6	5.3
A_0037	*N*-Carbamoylaspartic acid	Carbamoyl-Asp	93072	N.D.	0.2	2.2	1.4	0.6	2.0	21	26	24
A_0038	PRPP	PRPP	7339	8.8	34	48	4.7	6.3	21	72	53	52
A_0039	2-Phosphoglyceric acid	2-PG	439278	0.4	0.5	0.5	1.2	0.7	1.0	1.0	1.0	1.1
A_0040	2,3-Diphosphoglyceric acid	Diphosphoglycerate	186004	1.3	5.9	6.8	9.6	6.3	17	16	26	16
A_0041	3-Phosphoglyceric acid	3-PG	439183	3.5	3.7	4.1	8.5	4.3	8.4	8.5	8.2	8.3
A_0042	Phosphoenolpyruvic acid	PEP	1005	N.D.	0.6	0.9	1.6	N.D.	2.3	1.9	0.9	1.3
A_0043	GMP	GMP	6804	15	4.7	2.3	3.8	4.9	18	37	14	29
A_0044	AMP	AMP	6083	38	8.6	5.1	7.8	7.5	105	177	58	145
A_0045	2-Oxoisovaleric acid	2-KIV	49	N.D.	N.D.	N.D.	N.D.	N.D.	N.D.	5.5	4.0	6.6
A_0046	GDP	GDP	8977	13	8.4	6.3	22	19	53	103	50	88
A_0047	Lactic acid	Lactic acid	612	112	114	218	1,959	1,618	1,307	15,714	7,418	14,009
A_0048	ADP	ADP	6022	56	30	27	100	88	335	489	254	449
A_0049	GTP	GTP	6830	34	61	54	273	177	348	440	287	387
A_0050	Glyoxylic acid	Glyoxylic acid	760	N.D.	N.D.	N.D.	N.D.	N.D.	N.D.	N.D.	N.D.	N.D.
A_0051	ATP	ATP	5957	157	290	274	1,586	1,078	1,838	1,619	1,198	1,677
A_0052	Glycerol 3-phosphate	Glycerol 3-phosphate	439162	4.1	4.3	8.9	126	103	219	310	134	178
A_0053	Glycolic acid	Glycolic acid	757	50	23	19	51	115	24	23	14	N.D.
A_0054	Pyruvic acid	Pyruvic acid	1060	36	22	32	83	75	55	140	76	230
A_0055	*N*-Acetylglutamic acid	N-AcGlu	70914	N.D.	1.0	0.9	2.1	N.D.	2.9	1.8	2.0	3.1
A_0056	2-Hydroxyglutaric acid	2-Hydroxyglutaric acid	43	1.3	N.D.	0.4	2.2	2.7	1.2	5.2	3.6	7.1
A_0057	Carbamoylphosphate	Carbamoyl-P	278	N.D.	N.D.	N.D.	N.D.	N.D.	0.13	N.D.	N.D.	N.D.
A_0058	Succinic acid	Succinic acid	1110	15	7.3	8.6	22	33	26	94	52	93
A_0059	Malic acid	Malic acid	525	56	26	20	N.D.	N.D.	35	325	241	292
A_0060	2-Oxoglutaric acid	2-OG	51	N.D.	N.D.	N.D.	N.D.	N.D.	N.D.	63	22	30
A_0061	Fumaric acid	Fumaric acid	444972	1.6	N.D.	0.4	N.D.	N.D.	4.9	65	41	56
A_0062	Citric acid	Citric acid	311	8.4	50	63	116	124	82	425	286	243
A_0063	*cis*-Aconitic acid	cis-Aconitic acid	643757	N.D.	1.0	1.5	1.3	0.4	1.1	11	6.3	4.9
A_0064	Isocitric acid	Isocitric acid	1198	N.D.	N.D.	0.06	N.D.	N.D.	N.D.	16	8.8	3.3
C_0001	Urea	Urea	1176	N.D.	N.D.	N.D.	N.D.	N.D.	N.D.	N.D.	N.D.	N.D.
C_0002	Gly	Gly	750	145	222	212	1,697	1,410	2,199	1,507	1,041	2,141
C_0003	Putrescine	Putrescine	1045	N.D.	N.D.	N.D.	N.D.	N.D.	N.D.	N.D.	N.D.	N.D.
C_0004	Ala	Ala	602	25	116	136	766	315	430	800	330	1,098
C_0005	β-Ala	b-Ala	239	5.6	7.9	3.0	142	33	54	427	65	86
C_0006	Sarcosine	Sarcosine	1088	N.D.	N.D.	N.D.	N.D.	N.D.	N.D.	N.D.	N.D.	N.D.
C_0007	γ-Aminobutyric acid	g-Aminobutyric acid	119	15	8.4	3.1	12	N.D.	3.9	21	4.7	9.3
C_0008	*N*,*N*-Dimethylglycine	DMG	673	N.D.	N.D.	N.D.	N.D.	N.D.	N.D.	N.D.	N.D.	N.D.
C_0009	Choline	Choline	305	50	34	67	343	183	445	275	251	383
C_0010	Ser	Ser	617	30	60	82	138	138	159	108	134	607
C_0011	Carnosine	Carnosine	439224	1.1	2.7	1.4	0.3	N.D.	1.7	0.4	N.D.	N.D.
C_0012	Creatinine	Creatinine	588	1.3	2.6	1.5	3.2	N.D.	N.D.	4.2	N.D.	0.7
C_0013	Pro	Pro	614	42	129	138	358	144	842	1,783	956	1,904

	Compound name	Pathway Label	PubChem CID	Fresh			Bead			Beadless		
				Donor 1	Donor 2	Donor 3	Donor 1	Donor 2	Donor 3	Donor 1	Donor 2	Donor 3
C_0014	Betaine	Betaine	247	N.D.	N.D.	N.D.	N.D.	N.D.	N.D.	N.D.	N.D.	N.D.
C_0015	Val	Val	1182	N.D.	37	52	219	229	351	314	286	637
C_0016	Thr	Thr	6288	56	82	83	369	391	632	466	512	1,111
C_0017	Homoserine	Homoserine	12647	N.D.	N.D.	N.D.	N.D.	N.D.	N.D.	N.D.	N.D.	N.D.
C_0018	Betaine aldehyde	BTL	249	N.D.	N.D.	N.D.	N.D.	N.D.	N.D.	N.D.	N.D.	N.D.
C_0019	Cys	Cys	594	N.D.	N.D.	N.D.	N.D.	N.D.	N.D.	N.D.	2.3	5.1
C_0020	Hydroxyproline	Hydroxyproline	5810	N.D.	7.4	6.2	46	21	N.D.	35	N.D.	8.4
C_0021	Creatine	Creatine	586	23	11	15	34	2.0	4.1	61	3.3	3.9
C_0022	Ile	Ile	791	11	40	56	218	225	290	315	265	602
C_0023	Leu	Leu	857	14	42	59	207	213	262	293	248	574
C_0024	Asn	Asn	236	5.4	36	48	185	104	189	173	112	525
C_0025	Ornithine	Ornithine	389	1.4	1.3	N.D.	22	15	14	29	15	40
C_0026	Asp	Asp	424	783	978	436	1,977	1,975	2,289	358	518	1,437
C_0027	Homocysteine	Homocysteine	778	N.D.	N.D.	N.D.	N.D.	N.D.	N.D.	N.D.	N.D.	N.D.
C_0028	Adenine	Adenine	190	1.0	1.0	N.D.	N.D.	N.D.	N.D.	0.7	N.D.	N.D.
C_0029	Hypoxanthine	Hypoxanthine	790	N.D.	N.D.	N.D.	N.D.	N.D.	N.D.	2.6	N.D.	4.7
C_0030	Spermidine	Spermidine	1102	N.D.	N.D.	N.D.	N.D.	N.D.	4.0	2.4	1.5	2.2
C_0031	Gln	Gln	738	52	63	67	1,275	1,203	1,906	576	792	1,892
C_0032	Lys	Lys	866	55	62	45	175	181	171	145	127	376
C_0033	Glu	Glu	611	122	713	617	3,875	3,388	5,976	3,273	2,947	6,057
C_0034	Met	Met	876	5.0	14	18	39	48	55	53	38	94
C_0035	Guanine	Guanine	764	N.D.	N.D.	N.D.	N.D.	N.D.	N.D.	N.D.	N.D.	N.D.
C_0036	His	His	773	11	18	19	66	63	78	66	61	137
C_0037	Carnitine	Carnitine	85	17	11	17	N.D.	N.D.	N.D.	N.D.	N.D.	N.D.
C_0038	Phe	Phe	994	10	22	26	82	97	107	135	122	280
C_0039	Arg	Arg	6322	31	31	34	143	113	88	92	49	161
C_0040	Citrulline	Citrulline	9750	4.0	3.0	2.8	4.9	N.D.	N.D.	2.3	N.D.	N.D.
C_0041	Tyr	Tyr	1153	12	21	26	73	80	88	129	112	252
C_0042	*S*-Adenosylhomocysteine	SAH	439155	N.D.	N.D.	N.D.	N.D.	N.D.	N.D.	1.3	N.D.	N.D.
C_0043	Spermine	Spermine	1103	N.D.	N.D.	N.D.	N.D.	N.D.	13	9.3	5.5	6.2
C_0044	Trp	Trp	1148	N.D.	5.8	5.7	19	19	18	29	24	57
C_0045	Cystathionine	Cystathionine	834	N.D.	N.D.	N.D.	N.D.	N.D.	N.D.	N.D.	N.D.	3.4
C_0046	Adenosine	Adenosine	60961	N.D.	N.D.	N.D.	N.D.	N.D.	3.0	1.5	N.D.	N.D.
C_0047	Inosine	Inosine	6021	N.D.	N.D.	N.D.	N.D.	N.D.	N.D.	N.D.	N.D.	N.D.
C_0048	Guanosine	Guanosine	6802	N.D.	N.D.	N.D.	N.D.	N.D.	N.D.	N.D.	N.D.	N.D.
C_0049	Argininosuccinic acid	ArgSuccinate	16950	N.D.	N.D.	N.D.	N.D.	N.D.	N.D.	5.7	3.3	8.5
C_0050	Glutathione (GSSG)	GSSG	65359	115	132	80	189	377	151	424	774	694
C_0051	Glutathione (GSH)	GSH	124886	101	108	122	216	83	1,118	968	970	2,237
C_0052	*S*-Adenosylmethionine	SAM	34755	8.2	4.9	3.9	12	N.D.	8.9	9.8	9.3	27
-	Adenylate Energy Charge	No Label		0.7	0.9	0.9	1.0	1.0	0.9	0.8	0.9	0.8
-	Total Adenylate	No Label		251	328	307	1,694	1,173	2,277	2,285	1,510	2,271
-	Guanylate Energy Charge	No Label		0.7	0.9	0.9	1.0	0.9	0.9	0.8	0.9	0.9
-	Total Guanylate	No Label		62	74	62	298	201	419	580	352	504
-	GSH/GSSG	No Label		0.9	0.8	1.5	1.1	0.2	7.4	2.3	1.3	3.2
-	Total Glutathione	No Label		332	372	282	593	838	1,420	1,815	2,519	3,624
-	NADPH/NADP+	No Label		3.3	4.5	4.4	3.2	3.4	1.9	2.0	1.8	2.0
-	NADH/NAD+	No Label		1.2	0.7	0.6	0.09	0.14	0.06	0.09	0.09	0.10
	NAD+/NADH			0.9	1.3	1.7	11.3	7.0	15.7	11.1	11.6	10.2
-	Lactate/Pyruvate	No Label		3.1	5.1	6.7	24	22	24	112	98	61
-	Glycerol 3-phosphate/DHAP	No Label		1.0	1.4	13	N.A.	N.A.	122	217	67	39
-	Total Amino Acids	No Label		1,409	2,691	2,160	11,878	10,337	16,132	10,614	8,678	19,946
-	Total Essential Amino Acids	No Label		162	324	364	1,393	1,466	1,964	1,814	1,684	3,866
-	Total Non-essential Amino Acids	No Label		1,247	2,368	1,796	10,486	8,871	14,168	8,800	6,994	16,080
-	Total Glucogenic Amino Acids	No Label		1,340	2,587	2,056	11,496	9,943	15,698	10,177	8,303	18,997
-	Total Ketogenic Amino Acids	No Label		157	275	301	1,141	1,206	1,568	1,510	1,411	3,252
-	Total BCAA	No Label		25	120	167	643	667	903	921	798	1,812
-	Total Aromatic Amino Acids	No Label		22	48	58	173	196	213	293	259	590
-	Fischer's Ratio	No Label		1.2	2.5	2.9	3.7	3.4	4.2	3.1	3.1	3.1
-	Total Glu-related Amino Acids	No Label		258	954	875	5,716	4,911	8,891	5,790	4,806	10,151
-	Total Pyr-related Amino Acids	No Label		256	486	519	2,989	2,274	3,438	2,911	2,045	5,019
-	Total Acetyl CoA-related Amino Acids	No Label		80	150	166	618	639	741	781	664	1,609
-	Total Fumarate-related Amino Acids	No Label		22	42	52	154	176	196	263	235	532
-	Total Succinyl CoA-related Amino Acids	No Label		16	91	126	475	503	696	681	588	1,333
-	Total Oxaloacetate-related Amino Acids	No Label		788	1,013	483	2,161	2,079	2,478	532	630	1,962
-	Malate/Asp	No Label		0.07	0.03	0.05	N.A.	N.A.	0.02	0.9	0.5	0.2
-	Citrulline/Ornithine	No Label		2.9	2.4	N.A.	0.2	N.A.	N.A.	0.08	N.A.	N.A.
-	Glu/2-Oxoglutarate	No Label		N.A.	N.A.	N.A.	N.A.	N.A.	N.A.	52	132	205
-	G6P/R5P	No Label		1.2	2.2	2.1	4.6	3.0	0.7	2.2	7.9	2.5
-	SAM/SAH	No Label		N.A.	N.A.	N.A.	N.A.	N.A.	N.A.	7.4	N.A.	N.A.
-	Putrescine/Spermidine	No Label		N.A.	N.A.	N.A.	N.A.	N.A.	N.A.	N.A.	N.A.	N.A.

ID consists of analysis mode and number. 'C' and 'A' showed cation and anion modes, respectively.
N.D. (Not Detected): The target peak or metabolite was below detection limits.
N.A. (Not Available): The calculation was impossible because of insufficience of the data.¶
The data are sorted by ID in ascending order.
